# Association of blood pressure trajectories with coronary heart disease among the disabled population in Shanghai, China: a cohort study of 7 years following up

**DOI:** 10.1186/s40001-023-01240-1

**Published:** 2023-08-09

**Authors:** Yao Li, Jing Wu, Yiyan Wang, Hongmei Lei, Chenghua Jiang, Hua Zhai, Hengjing Wu

**Affiliations:** 1 Clinical Center for Intelligent Rehabilitation Research, Shanghai YangZhi Rehabilitation Hospital (Shanghai Sunshine Rehabilitation Center), School of Medicine, Tongji University, Shanghai, 201619 China; 2 Department of Fundamental Nursing, School of Nursing, Shanghai University of Traditional Chinese Medicine, Shanghai, 201203 China

**Keywords:** Coronary heart disease, Blood pressure trajectories, Disabled population, Cohort study

## Abstract

**Background:**

Much less is known about the importance of blood pressure (BP) trajectories concerning the incidence of coronary heart disease (CHD) in people with disabilities. Our aim was to evaluate this association.

**Methods:**

This cohort study surveyed 5711 adults from the Shanghai Disability Health Survey from June 2012 to June 2019. The latent class growth mixture model was used to examine distinct BP trajectories. We evaluated the association of BP trajectories with the risk of CHD by Cox proportional hazard models. The model for CHD risk fitted to BP trajectories was compared with models fitted to other BP-related indicators by goodness-of-fit, discrimination, and calibration.

**Results:**

During a median follow-up of 71.74 months, 686 cases (median age was 49.03 (54.49, 58.55) years, 51.90% female) with CHD were identified, with a cumulative incidence of 12.01%. Systolic BP (SBP) and diastolic BP (DBP) were categorized into three classes, respectively. A statistically significant association was only observed between SBP trajectories and CHD. Compared with the normotensive stable SBP group (*n* = 1956), the prehypertension-stable group (*n* = 3268) had a higher risk (adjust hazards ratio (aHR) = 1.266, 95% confidence interval (CI) 1.014–1.581), and the stage 1 hypertension-decreasing group (*n* = 487) had the highest risk (aHR = 1.609, 95%CI 1.157–2.238). Among the BP-related indicators, the SBP trajectory was the strongest predictor of new-onset CHD. Findings were similar when sensitivity analyses were conducted.

**Conclusions:**

SBP trajectory was a more important risk factor for CHD than other BP-related indicators and stringent BP control strategies may be effective for primary CHD prevention in the disabled population.

**Supplementary Information:**

The online version contains supplementary material available at 10.1186/s40001-023-01240-1.

## Background

According to the latest report on disability by the World Health Organization [1], more than one billion individuals, around 15% of the global population, lived with some form of disability. Meanwhile, a recent report indicated that the number of people with disabilities has reached nearly 7% of China's total population [2]. Studies have shown a strong link between the increased incidence of disability and chronic diseases, such as hypertension, diabetes, and stroke [3, 4]. In addition, the huge health disparity between people with and without disabilities has been widely demonstrated [5, 6]. People with disabilities may be more susceptible to various chronic diseases due to greater social isolation and less access to preventive care [7, 8]. However, few studies have analyzed the epidemic of the coronary heart disease (CHD) that accompany individuals who are already disabled.

Blood pressure (BP) is a leading treatable risk factor for CHD, a common chronic cardiovascular disease (CVD) that exists widely in the world, and has been deeply studied in the general population [9, 10]. Most studies focusing on the relationship between BP and the onset of CHD were mainly based on BP data from single measurements at baseline or the variability of multiple measurements [11–14]. Results from such approaches have suggested that the risk of incident CHD increases with increasing baseline BP and variability in BP. However, the above BP indicators may not adequately reflect longitudinal BP changes in individuals. Several recent studies have explored the role of BP patterns over time (i.e., BP trajectories) in predicting CVD outcomes, dynamically reflecting the impact of arterial BP on the development of diseases [15–19]. In particular, high-level and rapidly increasing BP patterns are associated with a high risk of stroke over an age range from 55 to 106 years. Nevertheless, much less is known about the importance of BP trajectories over time concerning the incidence of CHD in people with disabilities.

Thus, this study aimed to identify distinct longitudinal trajectories of systolic BP (SBP) and diastolic BP (DBP) over 7 years and to investigate the association of these trajectories with the risk of the onset of CHD among the disabled population. Moreover, we compared other BP-related indicators concerning the incident CHD.

## Methods

### Design and study populations

The cohort study comprised the adult disabled population who were newly diagnosed with CHD and derived from the Shanghai Disability Health Survey (SDHS) conducted by the Shanghai Disabled Persons' Federation. This ongoing SDHS was initiated in June 2012 and followed up annually to provide free health examination services to disabled people through designated medical institutions. Until June 2019, 10,505 potential individuals from 16 districts in Shanghai were recruited for the SDHS at Shanghai YangZhi Rehabilitation Hospital (Shanghai Sunshine Rehabilitation Center), one of the largest designated medical and healthcare institutions. Subjects who voluntarily participated in this program have informed and consented to the possible use of relevant health data for scientific research. The study has been ethically approved by the medical ethics committee of the institution (No. YZ 2019-051).

**Fig. 1:**
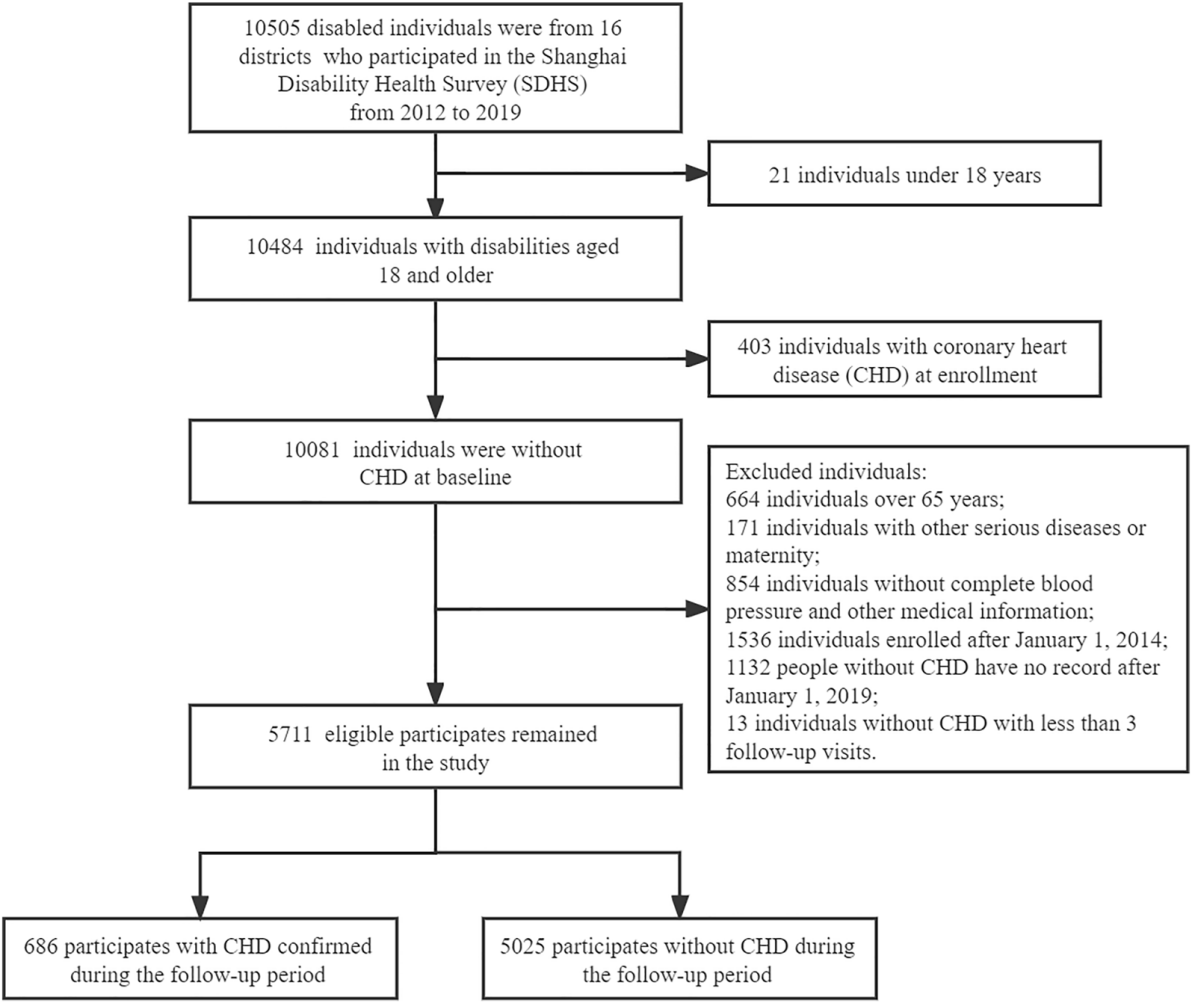
Consort flow diagram for participants included in the study

This study selected 5711 participants based on the exclusion criteria: (i) under 18 or over 65 years; (ii) with CHD at enrollment between 2012 and 2013; (iii) with other serious diseases, such as malignancy, organ failure, severe trauma, cortical damage; (iii) pregnant or lactating women, or those who are planning to become pregnant; (iv) failure to complete electronic health records; (v) less than 3 health examinations if CHD was not diagnosed in 2019, as shown in Fig. 1.

### Assessment of blood pressure

BP was measured by trained technicians using a cuffed electronic sphygmomanometer (Omron Corp., Tokyo, Japan) on participants after at least a 5-min rest, the upper arm with the higher reading was measured twice and the average was recorded in the electronic health system. For participants with missing upper limbs, the physician measured BP from both legs (ankle-posterior tibial artery) in the prone position. A total of 23 participants underwent leg BP measurements and the results were converted by a formula that was used to ensure consistency with arm measurements [20].

### Assessment of coronary heart disease

CHD was defined as fatal ischemic heart disease (International Classification of Disease 10th revision code, ICD-10 I20-I25) or non-fatal myocardial infarction (ICD-10 I21-I23). At baseline, the history of CHD was assessed by interview and verified using medical records. Of all potential CHD, medical records from general practitioners and hospital discharge diagnoses were collected and reviewed by physicians.

### Covariates

All participants had a medical and functional assessment by a medical professional. The health examination included anthropometry measurements (height, weight, resting BP, etc.), fasting biochemical indicators (blood, urine indicators, etc.), electrocardiogram, imaging ultrasound, as well as a questionnaire on demographic variables (gender, age, education, etc.), comorbidities (hypertension, diabetes, fatty liver disease, etc.) and disability condition (classification and grade). The classification and grade criteria of disabilities are based on the Chinese national standard for Classification and Grading Criteria of Disabilities (GB/T 26341-2010) [21]. Body mass index (BMI) was defined as the weight (kg)/(height (m)^2^), with the height measured in the supine position for those unable to stand.

Laboratory tests included red blood cell count (RBC), white blood cell count (WBC), platelet (PLT), hemoglobin (Hb), serum creatinine (SCr), serum urea (SU); uric acid (UA); fasting blood glucose (FBG), total cholesterol (TC), triglyceride (TG), total protein (TP), albumin (Alb), globulin (Glo), alpha fetoprotein (AFP), carcinoembryonic antigen (CEA), and alanine aminotransferase (Alt). The estimated glomerular filtration rate (eGFR) was calculated based on the Modification of Diet in the Renal Disease equation [22].

### Statistical analysis

Using the lcmm package in Statistical software R (version 4.2.1, R Core Team, R Foundation for Statistical Computing, Vienna, Austria), the latent class growth mixture model (LCGMM) was applied to identify longitudinal trajectories over time in the disabled population. We compared two- to five-class models iterating 1st- to 3rd-degree fractional polynomials. The best-fitting one was determined by the minimum absolute value of the Bayesian information criterion (BIC), the average posterior probability (AvePP) of each subgroup, the proportion of each subgroup with eligible posterior probability, and the membership of each subgroup. The age and gender of each wave were adjusted when the LCGMM was performed as relevant studies have shown that these variables may influence the evolution of BP [16, 23].

If distributed normally by the Kolmogorov–Smirnov test, continuous variables were presented as means and standard deviations; otherwise, medians and interquartile ranges were applied. Categorical variables were presented by numbers and proportions. Descriptive analyses were compared between the CHD group and non-CHD group using independent sample t-test for normally distributed data, Pearson *χ*^2^ test for categorical variables, and Mann–Whitney U test or Kruskal–Wallis test for non-parametric continuous data.

Baseline characteristics were compared between the different BP trajectory subgroups. The Kaplan–Meier method was performed to estimate the overall cumulative incidence of CHD from the baseline study visit onwards. Next, Cox proportional hazards models by survival package in R (version 4.2.1, R Core Team, R Foundation for Statistical Computing, Vienna, Austria) were used to investigate the association of BP trajectories, single BP (measured at baseline), average BP (average of all available BP levels during the follow-up), and coefficient of variation (CV) of BP (standard deviation/average BP during the follow-up) with risk of CHD by adjusting hazards ratio (aHR) and the corresponding 95% confidence interval (CI), respectively. Significant variables in the univariate model (*p* < 0.1) were entered into the multivariate model as covariates, including age, gender, education, etc. In addition, we calculated the population attributable risk (PAR). The akaike information criterion (AIC) and BIC of models were compared to investigate the best model fit. In addition, discrimination was measured using receiver operating characteristic (ROC) curves and the area under the curve (AUC), and calibration was measured using calibration plots. Sensitivity analyses were conducted to explore the robustness of our results in modeling without fitting age and sex and hypertension stratification subgroups. A 2-sided *P* < 0.05 was considered statistically significant. (see Additional file 1 for details).

## Results

### Baseline characteristics of all participants

A total of 5711 eligible individuals were included in this study, of which 686 cases had new-onset CHD during a median follow-up period of 71.74 months, with a cumulative incidence of 12.01%. The median age was 49.03 (54.49, 58.55) years and 2759 (48.31%) were female. More than half (55.86%) of the participants were physically disabled, and the highest percentage of disability grades were mild and moderate (76.88%). Baseline characteristics of the participant by CHD status are summarized in Additional file 2: Table S1. People with CHD were more likely to be older, female, higher educational level, fatter and physically disabled, and had a higher prevalence of hypertension and diabetes mellitus, as well as higher SBP compared to those with no CHD. Several metabolic biomarkers were also significantly different when compared between the two groups, including Hb, RBC, WBC, FBG, TC, TG, Alb, UA, SU, and eGFR (all *p* < 0.05).

### Characteristics of the BP trajectory classes

**Fig. 2:**
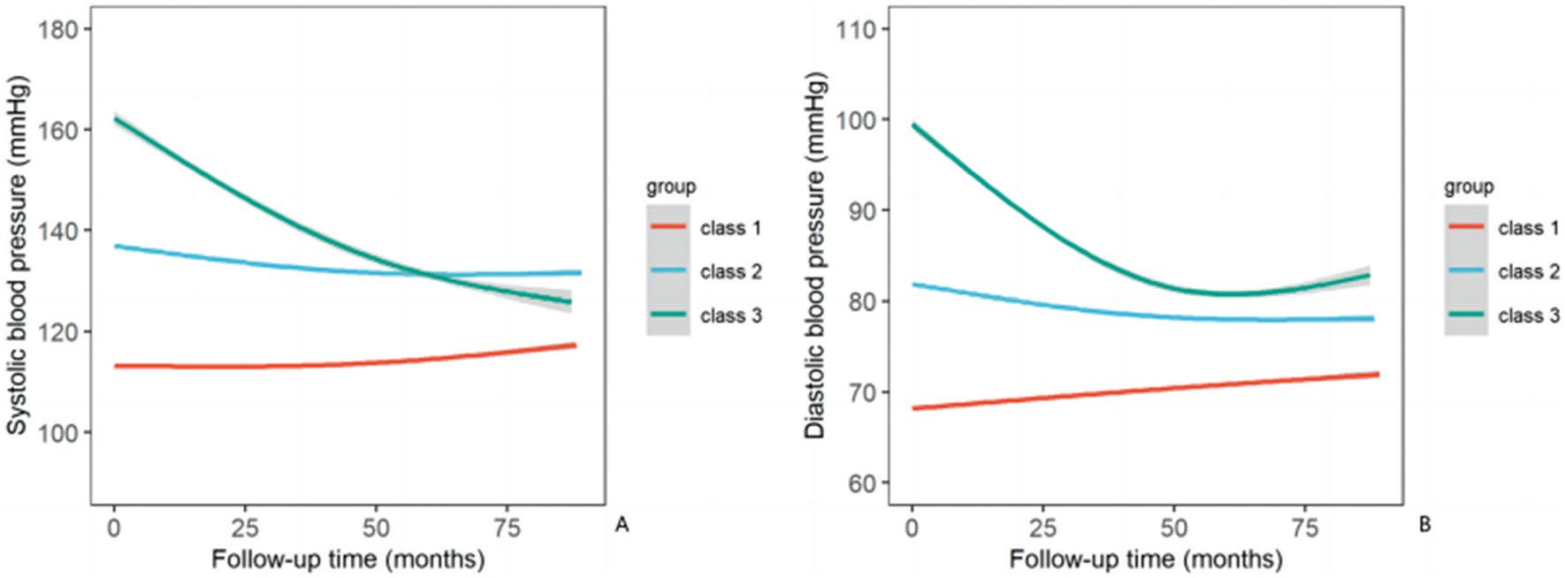
Trajectories of blood pressure predicted by the LCGMM among 5711 disabled individuals. **A**, SBP. **B**, DBP. The solid line represents the average BP in a class and the shaded area indicated 95% CIs

When investigating the trajectories, three categories of SBP and DBP were identified as the best fit with the lowest BIC, suitable AvePP, and a sufficient number of each subgroup, respectively (Additional file 2: Tables S2 and S3). Figure 2A shows the three trajectories of SBP based on the LCGMM. The largest class was characterized by a sustained slow decline in prehypertensive SBP, starting at ≈136 mmHg at enrollment and declining to ≈130 mm Hg at the end (class 2, *n* = 3268). The smallest class was characterized by a much steeper decrease in high-level SBP from approximately 161 to 129 mmHg during the follow-up (class 3, *n* = 487). In addition, another class was characterized by a sustained slow rise in normotensive SBP from approximately 122 to 129 mmHg (class 1, *n* = 1956). Figure 2B presents the three trajectories of DBP, similar to that of SBP, but the smallest class drops rapidly from 100 to 82 mmHg and then rises slowly (class 3, *n* = 534). In addition, the shape of trajectories for the other indicators (FBG, TC, TG, and eGFR) was shown in Additional file 2: Figure S1.

Demographic and health-related characteristics across BP trajectory groups were described in Table 1. Statistically significant differences were detected among SBP classes in age, gender, education level, BMI, classification of disabilities, comorbidities (except hyperlipemia), BP (SBP and DBP), and some metabolic biomarkers (FBG, TC, TG, etc.) (all *p* < 0.05). Characteristics with statistically significant differences among DBP trajectory groups were almost identical to those for SBP. During the follow-up period, longitudinal BP and some metabolic indicators (FBG, TC, TG, and eGFR) of the disabled population by BP trajectories and CHD status were displayed in Additional file 2: Tables S4 and S5, respectively. There were both significant differences in five average indicators (SBP, DBP, FBG, TC, and TG), three CV indicators (SBP, DBP, and FBG), and trajectories of four metabolic biomarkers among different SBP and DBP trajectory groups (all *p* < 0.05). Compared to the group without CHD, disabled people with CHD had higher average SBP and FBG, higher CVs for SBP, DBP, FBG, TC, and eGFR, as well as different proportions of trajectories for SBP, DBP, FBG, TC, and eGFR (all *p* < 0.05).

**Table 1:** Baseline characteristics of 5711 disabilities by BP trajectory cluster

Characteristics	SBP				DBP			
	Class 1 *n* = 1956	Class 2 *n* = 3268	Class 3 *n* = 487	*P*-value	Class 1 *n* = 1671	Class 2 *n* = 3506	Class 3 *n* = 534	*P*-value
Age, yrs	43.86 (52.20,56.67)	48.87 (54.32,58.66)	49.43 (55.42,59.49)	< 0.001	44.03 (52.54,57.35)	48.45 (54.11,58.43)	49.03 (54.49,58.55)	< 0.001
Female, *n* (%)	1011 (51.69)	1510 (46.21)	238 (48.87)	0.001	895 (53.56)	1641 (46.81)	223 (41.76)	< 0.001
Education, *n* (%)				0.001				0.191
Primary school & Illiterate	332 (16.97)	640 (19.58)	120 (24.64)		305 (18.25)	670 (19.11)	117 (21.91)	
Junior high school	1507 (77.04)	2463 (75.37)	347 (71.25)		1266 (75.76)	2656 (75.76)	395 (73.97)	
Senior high school & Higher	117 (5.98)	165 (5.05)	20 (4.11)		100 (5.98)	180 (5.13)	22 (4.12)	
BMI, kg/m^2^	22.71 ± 3.15	24.61 ± 3.40	25.71 ± 3.79	< 0.001	22.60 ± 3.21	24.48 ± 3.39	25.79 ± 3.58	< 0.001
Classification of disabilities, *n* (%)				< 0.001				< 0.001
Intellectual & mental disability	371 (18.97)	502 (15.36)	76 (15.61)		347 (20.77)	526 (15.00)	76 (14.23)	
Hearing & speech disability	143 (7.31)	178 (5.45)	21 (4.31)		121 (7.24)	199 (5.68)	22 (4.12)	
Visual disability	423 (21.63)	712 (21.79)	95 (19.51)		330 (19.75)	781 (22.28)	119 (22.28)	
Physical disability	1019 (52.10)	1876 (57.41)	295 (60.57)		873 (52.24)	2000 (57.05)	317 (59.36)	
Grading of disabilities, *n* (%)				0.203				0.487
Very severe disability	167 (8.54)	235 (7.19)	35 (7.19)		143 (8.56)	254 (7.24)	40 (7.49)	
Severe disability	293 (14.98)	508 (15.54)	82 (16.84)		270 (16.16)	526 (15.00)	87 (16.29)	
Moderate disability	632 (32.31)	1130 (34.58)	148 (30.39)		551 (32.97)	1190 (33.94)	169 (31.65)	
Mild disability	864 (44.17)	1395 (42.69)	222 (45.59)		707 (42.31)	1536 (43.81)	238 (44.57)	
Comorbidities, *n* (%)								
Hypertension	130 (6.65)	1102 (33.72)	296 (60.78)	< 0.001	115 (6.88)	1093 (31.18)	320 (59.93)	< 0.001
Diabetes mellitus	138 (7.06)	462 (14.14)	94 (19.30)	< 0.001	142 (8.50)	457 (13.03)	95 (17.79)	< 0.001
Hyperlipemia	319 (16.31)	555 (16.98)	69 (14.17)	0.283	287 (17.18)	574 (16.37)	82 (15.36)	0.577
Fatty liver disease	158 (8.08)	336 (10.28)	45 (9.24)	0.031	120 (7.18)	374 (10.67)	45 (8.43)	< 0.001
Chronic kidney disease	152 (7.77)	303 (9.27)	77 (15.81)	< 0.001	119 (7.12)	327 (9.33)	86 (16.10)	< 0.001
Resting heart rate, beats/min	72 (76,84)	72 (76,84)	76 (82,88)	< 0.001	72 (76,84)	72 (76,84)	76 (80,84)	< 0.001
Blood pressure, mmHg								
SBP	113.08 ± 10.07	138.87 ± 12.46	167.58 ± 14.79	< 0.001	115.18 ± 13.32	136.25 ± 15.18	161.93 ± 17.57	< 0.001
DBP	68.63 ± 8.72	82.58 ± 9.32	95.23 ± 10.40	< 0.001	65.80 ± 7.28	81.93 ± 7.28	99.80 ± 8.53	< 0.001
Metabolic biomarkers								
AFP, ng/mL	0.40 (0.87,1.65)	0.45 (0.91,1.72)	0.50 (1.03,1.73)	0.020	0.41 (0.85,1.58)	0.45 (0.92,1.74)	0.48 (0.95,1.79)	0.005
CEA, ng/ml	0.43 (0.94,1.95)	0.53 (1.11,2.15)	0.59 (1.23,2.35)	< 0.001	0.45 (0.98,1.96)	0.51 (1.09,2.13)	0.53 (1.19,2.42)	< 0.001
Hb, g/L	136.24 ± 16.12	140.04 ± 16.00	141.89 ± 15.41	< 0.001	135.32 ± 15.85	139.82 ± 16.11	144.05 ± 14.68	< 0.001
RBC, 10^12^/L	4.49 ± 0.44	4.60 ± 0.45	4.66 ± 0.42	< 0.001	4.46 ± 0.44	4.59 ± 0.45	4.71 ± 0.43	< 0.001
WBC, 10^9^/L	6.09 ± 1.58	6.40 ± 1.57	6.76 ± 1.73	< 0.001	6.03 ± 1.51	6.39 ± 1.60	6.78 ± 1.65	< 0.001
PLT, 10^9^/L	198.79 ± 53.49	204.04 ± 55.16	211.67 ± 55.66	< 0.001	199.57 ± 53.58	203.58 ± 55.17	208.77 ± 54.97	0.002
FBG, mmol/L	4.70 (5.00,5.40)	4.90 (5.20,5.70)	5.10 (5.40,6.10)	< 0.001	4.70 (5.00,5.40)	4.90 (5.20,5.70)	5.00 (5.40,6.10)	< 0.001
TC, mmol/L	4.67 ± 0.88	4.87 ± 0.94	5.04 ± 0.98	< 0.001	4.67 ± 0.90	4.85 ± 0.93	5.04 ± 1.00	< 0.001
TG, mmol/L	0.79 (1.08,1.54)	0.93 (1.32,1.94)	1.06 (1.48,2.20)	< 0.001	0.78 (1.05,1.48)	0.93 (1.32,1.94)	1.05 (1.47,2.18)	< 0.001
Glo, g/L	27.96 ± 3.86	28.46 ± 3.69	29.56 ± 3.81	< 0.001	27.96 ± 3.78	28.43 ± 3.77	29.35 ± 3.66	< 0.001
Alt, U/L	14.00 (18.00,26.00)	16.00 (21.00,31.00)	17.00 (23.00,35.00)	< 0.001	14.00 (18.00,25.00)	16.00 (21.00,31.00)	17.00 (24.00,35.25)	< 0.001
TP, g/L	71.74 ± 4.09	72.47 ± 4.06	73.57 ± 4.12	< 0.001	71.67 ± 4.04	72.44 ± 4.12	73.49 ± 3.90	< 0.001
Alb, g/L	43.80 ± 2.28	44.01 ± 2.39	44.02 ± 2.53	0.006	43.71 ± 2.32	44.01 ± 2.35	44.15 ± 2.53	< 0.001
UA, μmol/L	304.01 ± 79.84	328.56 ± 87.01	341.93 ± 91.47	< 0.001	300.19 ± 78.24	327.64 ± 87.21	345.60 ± 88.19	< 0.001
SU, mmol/L	4.10 (4.90,5.80)	4.20 (4.90,5.90)	4.20 (5.10,5.90)	0.037	5.04 ± 1.29	5.09 ± 1.31	5.20 ± 1.62	0.043
SCr, μmol/L	50.30 (60.65,72.08)	50.70 (62.20,72.70)	49.50 (60.90,72.00)	0.137	49.70 (60.00,70.90)	50.60 (62.10,72.73)	51.38 (62.65,75.30)	< 0.001
eGFR, ml/min/1.73 m^2^	96.53 (112.86,135.92)	94.07 (112.33,133.90)	94.69 (111.88,134.06)	0.170	97.27 (113.61,136.59)	94.1 (112.34,134.26)	91.63 (110.98,131.93)	0.005

*BMI* body mass index, *AFP* alpha fetoprotein, *CEA* carcinoembryonic antigen, *FBG* fasting plasma glucose, *TC* total cholesterol, *TG* total triglyceride, *TP* total protein, *Alb* albumin, *Glo* globulin, *Alt* alanine aminotransferase, *UA* uric acid, *SCr* serum creatinine, *SU* serum urea, *Hb* hemoglobin, *RBC* red blood count, *WBC* white blood count, *PLT* platelet count, *eGFR* estimated glomerular filtration rate

### Association of BP trajectories with incident CHD

**Fig. 3:**
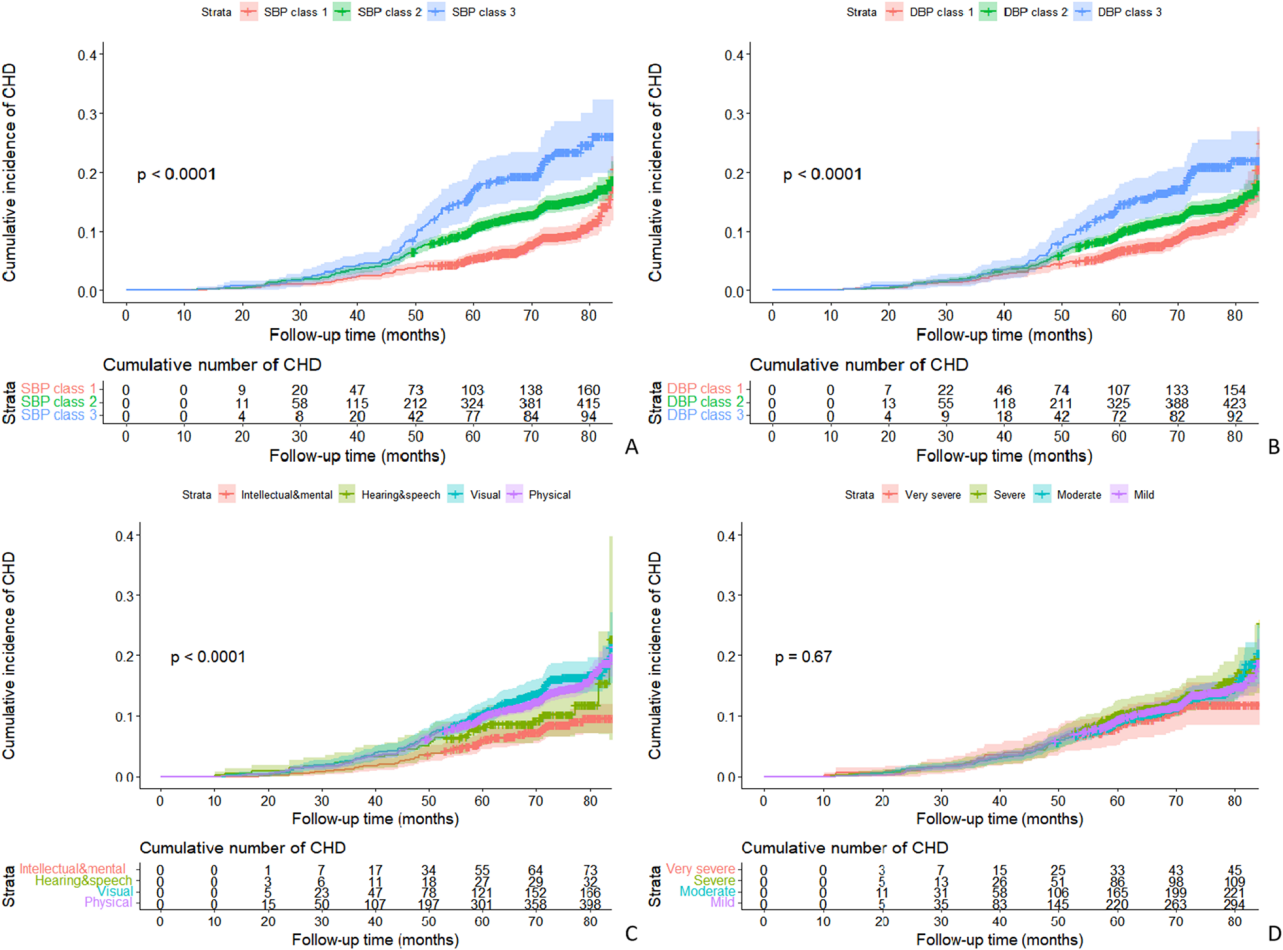
Kaplan–Meier curves for the overall cumulative incidence of CHD and number at risk, stratified by **A** SBP trajectories, **B** DBP trajectories, **C** classification of disabilities, **D** grading of disabilities. The shaded area indicates the range of 95% CIs for the corresponding cumulative incidence curve. P-value indicates the significance level from the comparison of incidence curves using the Log-rank test

Kaplan–Meier curves for the overall cumulative incidence and number at risk for CHD stratified by BP trajectories and disability-related indicators (classification and grade) are displayed in Fig. 3. Analysis showed significant differences in the cumulative incidence curves for SBP trajectories, DBP trajectories, and the classification (all *P* < 0.001). Furthermore, the variables that differed significantly in univariate Cox analysis (*p* < 0.1) were subsequently entered into each of the four multivariate models to assess the risk factors correlated with new-onset CHD (Additional file 2: Tables S6, S7, and S8). Significant differences were detected among SBP trajectories after adjustment for covariates listed above, with a significant increase in CHD risk for class 2 (aHR = 1.266, 95% CI 1.014–1.581) and class 3 (aHR = 1.609, 95% CI 1.157–2.238) relative to class 1, respectively (Table 2). Compared with the normotensive stable SBP group (class 1), the PAR was 12.89% in the prehypertension-stable group (class 2) and 5.24% in the stage 1 hypertension-decreasing group (class 3). However, the DBP trajectory was not statistically significant in the multivariate model.

**Table 2:** Association between BP-related indicators with incident CHD in the disabled population

Variables	Model 1^#^	Model 2^&^	Model 3^$^	Model 4^@^
SBP trajectories				
Class 1	Reference			
Class 2	1.266 (1.014, 1.581)^*^			
Class 3	1.609 (1.157, 2.238)^*^			
DBP trajectories				
Class 1	Reference			
Class 2	0.929 (0.719, 1.199)			
Class 3	1.062 (0.764, 1.475)			
SBP index		1.006 (1.001, 1.012)^*^	10.454 (1.407, 77.666)^*^	1.004 (1.000, 1.008)^*^
DBP index		–	4.652 (0.579, 37.409)	0.995 (0.984, 1.006)

^#^BP trajectories in the multivariate cox regression model 1 adjusted by age, gender, education level, classification of disabilities, hypertension, diabetes mellitus, SBP, DBP, FBG, TC, and eGFR at baseline, as well as trajectories of FBG, TC, and eGFR during follow-up

^&^Average SBP in the multivariate cox regression model 2 adjusted by baseline characteristics as in model 1, as well as average FBG and eGFR during follow-up

^$^Coefficient of variations (CVs) of BP in the multivariate cox regression model 3 adjusted by baseline characteristics as in model 1, as well as CVs of FBG, TC, and eGFR during follow-up

^@^BP at baseline in the multivariate cox regression model 4 adjusted by age, gender, education level, classification of disabilities, hypertension, diabetes mellitus, FBG, TC, and eGFR at baseline

^*^P < 0.05

In addition to the SBP trajectory, CHD risk was also associated with average SBP (aHR = 1.006, 95% CI 1.001, 1.012), CV of SBP (aHR = 10.454, 95% CI 1.407, 77.666), and baseline SBP (aHR = 1.004, 95% CI 1.000–1.008) by model 2 to model 4 analysis, respectively (Table 2). Comparing the evaluation indexes of four models, the SBP trajectory was more likely to be the strongest predictor of new-onset CHD in the disabled population (by the highest AUC and lowest BIC/AIC in Table 3, Additional file 2: Figures S2, S3).

**Table 3:** Comparison of evaluation indexes of four Cox proportional hazards models

Indexes	Model 1^#^	Model 2^&^	Model 3^$^	Model 4^@^
Goodness-of-fit				
AIC	2302.63	2324.38	2310.18	2330.04
BIC	2375.79	2384.23	2383.33	2389.89
Discrimination				
AUC	0.727^*^	0.709	0.713	0.706

^#^The trajectories of BP and metabolic biomarkers during follow-up were analyzed in model 1

^&^The average BP and metabolic biomarkers during follow-up were analyzed in model 2

^$^The CVs of BP and metabolic biomarkers during follow-up were analyzed in model 3

^@^The BP and metabolic biomarkers at baseline were analyzed in model 4

^*^P-values indicate that the significance level of the difference between the AUC of model 1 and the other three models were less than 0.05

### Sensitivity analyses

Without fitting age and sex in the LCGMM, the BP trajectories were in line with the main analyses (Additional file 2: Figure S4). The association of BP trajectories with new-onset CHD and comparison of four Cox models based on different BP-related indicators are presented in Additional file 2: Tables S9 and S10, which did not provide additional insights.

Another sensitivity assessment was performed to model the BP trajectory based on stratification of whether the individuals with disabilities had hypertension at baseline. Although both SBP and DBP trajectories were one less in the hypertensive subgroup than in the non-hypertensive subgroup (Additional file 2: Figures S5 and S6), the relationship between BP trajectories and CHD and the comparison of modeling results for the four BP-related metrics were similar to the primary results (Additional file 2: Tables S9 and S10).

## Discussion

Although previous studies have discussed the relationship between BP trajectories and CVDs risk in the general population, the association among the disabled population has rarely been elucidated. In the current study, we explored the association between BP trajectories and CHD risk in the disabled population using a substantial population-based cohort. First, the trajectories of SBP and DBP were categorized into three classes by LCGMM, respectively. Second, the disabled individuals in stage 1 hypertension-decreasing SBP group and prehypertension-stable SBP group had higher CHD risk relative to the normotensive-stable SBP group. Third, the SBP trajectory may be a stronger predictor of CHD than single baseline SBP, average SBP, and CV of SBP.

Our findings identified three SBP and DBP trajectories separately, these overall trend waves were consistent with previous cohort studies on BP trajectories associated with CVDs during follow-up in the general population but the number of trajectories ranged from three to five classes [15, 24, 25]. The main difference is that we did not find a trajectory of BP that rose significantly from normal levels to high values, which may be due to population differences and limitations of the LCGMM fitting subgroup sample. In addition, subgroup analysis revealed almost identical trajectories to the primary results in the no-hypertension group, but only two BP trajectories were found in the hypertensive group without the normotensive-stable BP class (< 130/80 mmHg).

The relationship between BP trajectories and CVD is not identical due to the heterogeneity in the characteristics of trajectories and populations in relevant studies. Our study suggests that the disabled individuals in the stage 1 hypertension-decreasing SBP group (160–120 mmHg) and the prehypertension-stable group (130–140 mmHg) may both increase the risk of future CHD, although a statistically significant association was not observed between DBP trajectories and CHD after multivariate adjustment. Among the general population, the Framingham Heart Study explored 16-year trajectories of BP and its association with the risk of atrial fibrillation in 4351 participants and found that the risk was doubled in participants who had initially elevated levels of SBP and then either decreased or increased SBP, yet the trajectory of DBP was not an independent predictor [18]. A 7-year cohort study determined that class 2 (SBP persistently around 133 mmHg) and class 3 (initially elevated SBP decreasing to normal) were associated with increased CVD risk relative to class 1 (initially normal levels increasing to above 140 mmHg) among 4067 individuals [15]. In China, five distinct BP trajectories over 4 years were identified in the Kailuan Study, the prehypertension-stable SBP group had higher stroke risk and individuals in the stage 2 hypertension-stable group (175–179 mmHg) had the highest risk of intracerebral hemorrhage and cerebral infarction relative to the normotensive-stable group (< 130 mmHg), and trajectories of DBP was also significantly associated with the risks of above outcomes [26]. Moreover, a meta-analysis among 123 studies with 613,815 participants revealed that lowering SBP to less than 130 mmHg significantly reduced the risk of CHD [11]. Our results indicated that people with disabilities may have an impact on health outcomes once they are chronically exposed to pre-hypertension or poorly controlled BP over their life course, and a more stringent BP control strategy may be more effective for primary CHD prevention. However, the optimal BP goal for primary CHD prevention remains unclear in the disabled population.

The association between single baseline BP as well as long-term variability and CHD risk have been previously demonstrated [9, 11–13, 27]. These findings were also revealed in our study, in addition to the fact that SBP trajectory was considered as an independent better predictor among disabled subjects. More information was added to the existing knowledge regarding risk factors and prevention of CHD as they suggest that effects of elevated BP levels may take several years to appear, and that longitudinal analysis of risk factors can improve the prediction of outcomes compared to single measurements or averages [26, 28]. Group-based trajectory modeling takes into account the averages and variations of multiple measurements to distinguish dynamic exposure in BP over time [29], which could reflect the potential impact of long-term BP on target organs. Available evidence showed that consecutive years of hypertensive trajectories are strongly correlated with subclinical atherosclerosis, intima-media thickness, and left ventricular mass index [30, 31]. Assessing BP trajectories provided a more nuanced understanding of the association between BP and CHD and improved prediction of CVD, our findings could help provide new insights into the prevention and management of CVDs in the disabled population.

Our study has several limitations. First, due to difficulties in collecting self-reported data from disabled people in the early study, we were unable to assess information on their diet, physical activity, the accessibility of medical resources, socioeconomic status, and care for them. However, all SDHS participants have access to Shanghai Disability Living Guarantee, which can reduce this gap to some extent. Second, low/high density lipoprotein cholesterol tests were not included in the examination due to limited funding for SDHS. Therefore, we attempted to mitigate this effect by entering trajectories of other objective indicators affecting BP in the multivariate model. Third, the lack of detailed information on antihypertensive treatment may lead to non-differential misclassification, but a sensitivity analysis stratified by hypertension was performed. Fourth, the subjects in this study may have overrepresented those who lived longer, because participants with poor cardiovascular profiles may have died before their next physical examination, which may have led to an underestimation of the association between BP trajectory and CHD risk.

## Conclusion

Our study observed three distinct BP trajectories in people with disabilities and these trajectories were associated with future CHD risk. Monitoring BP trajectories over time could provide a more important approach to identifying these individuals with higher risk and preventing CHD. Future research needs to explore the optimal BP goal for primary CVDs prevention in the disabled population.

### Supplementary Information

**Additional file 1. **Detailed description of statistical methods.

**Additional file 2.** eFigures and eTables.

## Data Availability

The data sets used and/or analyzed during the current study are available from the corresponding author on reasonable request.

## Abbreviations

BP: Blood pressure
CHD: Coronary heart disease
SBP: Systolic blood pressure
DBP: Diastolic blood pressure
HR: Hazard ratio
aHR: Adjust hazard ratio
CI: Confidence interval
CVD: Cardiovascular disease
SDHS: Shanghai disability health survey
BMI: Body mass index
RBC: Red blood cell count
WBC: White blood cell count
PLT: Platelet
Hb: Hemoglobin
SCr: Serum creatinine
SU: Serum urea
UA: Uric acid
FBG: Fasting blood glucose
TC: Total cholesterol
TG: Triglyceride
TP: Total protein
Alb: Albumin
Glo: Globulin
AFP: Alpha fetoprotein
CEA: Carcinoembryonic antigen
Alt: Alanine aminotransferase
eGFR: Estimated glomerular filtration rate
LCGMM: Latent class growth mixture model
PAR: Population attributable risk
BIC: Bayesian information criterion
AvePP: Average posterior probability
CV: Coefficient of variation
AIC: Akaike information criterion
ROC: Receiver operating characteristic curves
AUC: Area under the curve
