# Supplementary material for: Association of blood pressure trajectories with coronary heart disease among the disabled population in Shanghai, China: a cohort study of 7 years following up
Source: Eur J Med Res. 2023 Aug 9;28:275. doi: 10.1186/s40001-023-01240-1 (PMC10410884; doi:10.1186/s40001-023-01240-1)
Supplement: Supplementary file 1 — Additional file 1. Detailed description of statistical methods. [file 40001_2023_1240_MOESM1_ESM.docx]

**Statistical Analysis**

Using the lcmm package in Statistical software R (version 4.2.1, R Core Team, R Foundation for Statistical Computing, Vienna, Austria), the latent class growth mixture model (LCGMM) was applied to model BP (SBP and DBP) and some metabolic biomarkers (FBG, TC, TG, and eGFR) longitudinal trajectories over the follow-up period in the disabled population and to identify distinct subgroups following similar patterns. We compared two- to five-class LCGMM models iterating 1st- to 3rd-degree fractional polynomials. The best-fitting one was determined by the minimum absolute value of the bayesian information criterion (BIC), the average posterior probability (AvePP) of each subgroup not less than 0.7, the proportion of each subgroup with posterior probability greater than 0.7 not less than 0.65, and the membership of each subgroup not less than 5% of the total population. The age and gender of each wave were adjusted when the LCGMM was performed as relevant studies have shown that these variables may influence the evolution of BP.

If distributed normally by the Kolmogorov-Smirnov test, continuous variables were presented as means and standard deviations; otherwise, medians and interquartile ranges were applied. Categorical variables were presented by numbers and proportions. Descriptive analyses were compared between the CHD group and non-CHD group using independent sample t-test for normally distributed data, Pearson χ^2^ test for categorical variables, and Mann-Whitney U test or Kruskal-Wallis test for non-parametric continuous data.

Baseline characteristics were compared between subjects within the different estimated BP trajectory classes using one-way ANOVA for continuous variables if distributed normally or Kruskal–Wallis tests if not, and using chi-square tests or Fisher’s exact tests for categorical variables. Next, the Kaplan-Meier method was performed to estimate the overall cumulative incidence of CHD from the baseline study visit onwards. The log-rank test was used to compare the cumulative incidence curve for sub-populations, stratified by BP trajectories and disability-related indicators (classification and grade). Cox proportional hazards models by survival package in R (version 4.2.1, R Core Team, R Foundation for Statistical Computing, Vienna, Austria) were used to investigate the association of longitudinal trajectories BP, single BP (BP measured at baseline), average BP (average of all available BP levels during the follow-up), and coefficient of variation (CV) of BP (standard deviation/average BP during the follow-up) with risk of CHD by adjusting hazards ratio (aHR) and the corresponding 95% confidence interval (CI), respectively. All covariates of the Cox models satisfied the proportional hazards assumption, and their variance inflation factors were less than 5. Significant variables in the Univariate cox model (P < 0.1) were entered into the multivariate model as covariates, including age, gender, education, etc. Additionally, we calculated the population attributable risk (PAR): $PAR=p_{\text{exposure}}\times\frac{\text{a}HR-1}{\text{a}HR}\times100\%$, aHR were estimated from the model 1, and p_exposure_ represented the proportion of exposed individuals in patients with new-onset CHD. The akaike information criterion (AIC) and BIC of models were compared to investigate the best model fit. In addition, discrimination was measured using receiver operating characteristic (ROC) curves and the area under the curve (AUC), and calibration was measured using calibration plots. A 2-sided P value of <0.05 was considered statistically significant.

**Sensitivity Analyses**

Sensitivity analyses were conducted to explore the robustness of our results in modeling decisions and hypertension stratification subgroups. First, age and sex were not adjusted at each wave when performing the LCGMM to test the robustness of our observed association between BP trajectories and CHD. Then, due to the lack of detailed information (medication use and years of disease) about hypertensive patients, we wanted to understand whether the identified trajectories were consistent within the groups with and without hypertension at baseline.
