# Supplementary material for: Association of blood pressure trajectories with coronary heart disease among the disabled population in Shanghai, China: a cohort study of 7 years following up
Source: Eur J Med Res. 2023 Aug 9;28:275. doi: 10.1186/s40001-023-01240-1 (PMC10410884; doi:10.1186/s40001-023-01240-1)
Supplement: Supplementary file 2 — Additional file 2. eFigures and eTables. [file 40001_2023_1240_MOESM2_ESM.docx]

**Supplementary appendix**

This appendix has been provided by the authors to give readers additional information about their working figures and tables.

**CONTENT**

[eFigure 1. Trajectories of metabolic biomarkers predicted by the LCGMM among 5711 disabled individuals. A, FBG. B, TG. C, TG. D, eGFR. The solid line represents the average blood pressure in a class and the shaded area indicated 95% CIs 1](#_Toc13536)

[eFigure 2. ROC curves between four Cox proportional hazards models. P-value indicates the significance level from the comparison of the AUC difference between the two ROC curves 2](#_Toc21005)

[eFigure 3. Calibration plots of four Cox proportional hazards models. A, Model 1. B, Model 2. C, Model 3. D, Model 4 3](#_Toc10364)

[eFigure 5. Trajectories of BP and metabolic biomarkers predicted by the LCGMM among 1528 disabled individuals with hypertension at baseline. A, SBP, B, DBP, C, FBG. D, TG. E, TG. F, eGFR. The solid line represents the average blood pressure in a class and the shaded area indicated 95% CIs 5](#_Toc16127)

[eFigure 6. Trajectories of BP and metabolic biomarkers predicted by the LCGMM among 4183 disabled individuals without hypertension at baseline. A, SBP, B, DBP, C, FBG. D, TG. E, TG. F, eGFR. The solid line represents the average blood pressure in a class and the shaded area indicated 95% CIs 6](#_Toc754)

[eTable 1. Baseline characteristics of 5711 disabilities with CHD and No-CHD group 7](#_Toc25038)

[eTable 2. BIC values for different link functions and degrees of different variables 9](#_Toc16783)

[eTable 3. Posterior probabilities of latent classes with different variables 12](#_Toc30723)

[eTable 4. Longitudinal characteristics during follow-up of 5711 disabilities by BP trajectory cluster 14](#_Toc13170)

[eTable 5. Longitudinal characteristics during follow-up of 5711 disabilities with CHD and no-CHD group 16](#_Toc1269)

[eTable 6. Cox regression model for BP trajectories and CHD risk in the disabled population 18](#_Toc25703)

[eTable 7. Univariate Cox regression model for BP and CHD risk in the disabled population 20](#_Toc27596)

[eTable 8. Multivariate Cox regression model for blood pressure and CHD risk in the disabled population 21](#_Toc16224)

[eTable 9. Sensitivity analysis of the correlation between BP-related indicators and the incidence of CHD in the disabled population 23](#_Toc13965)

[eTable 10. Sensitivity analysis for the comparison of evaluation indicators of the four Cox proportional hazards models 25](#_Toc11705)

**
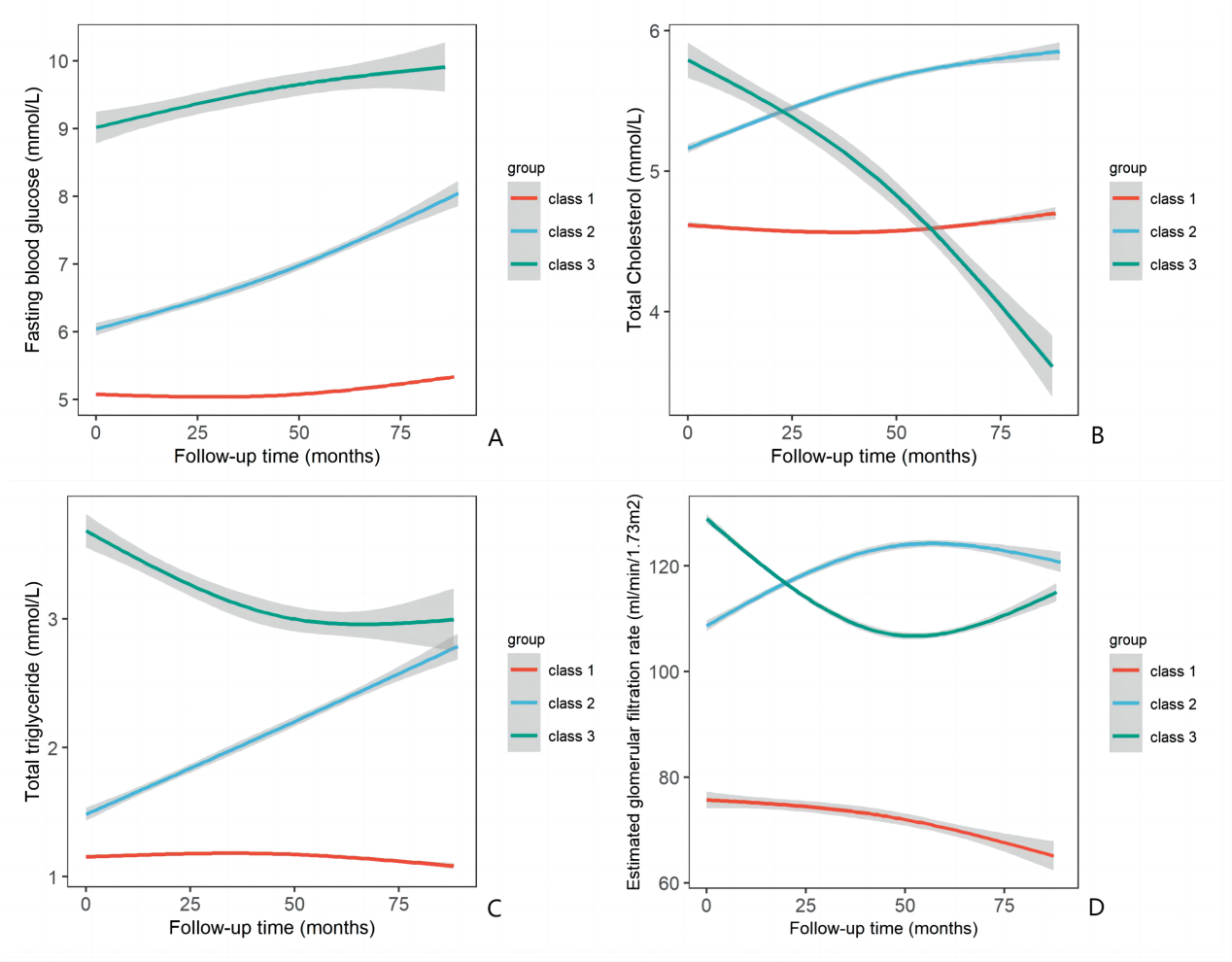
**

# eFigure 1. Trajectories of metabolic biomarkers predicted by the LCGMM among 5711 disabled individuals. A, FBG. B, TG. C, TG. D, eGFR. The solid line represents the average blood pressure in a class and the shaded area indicated 95% CIs

**
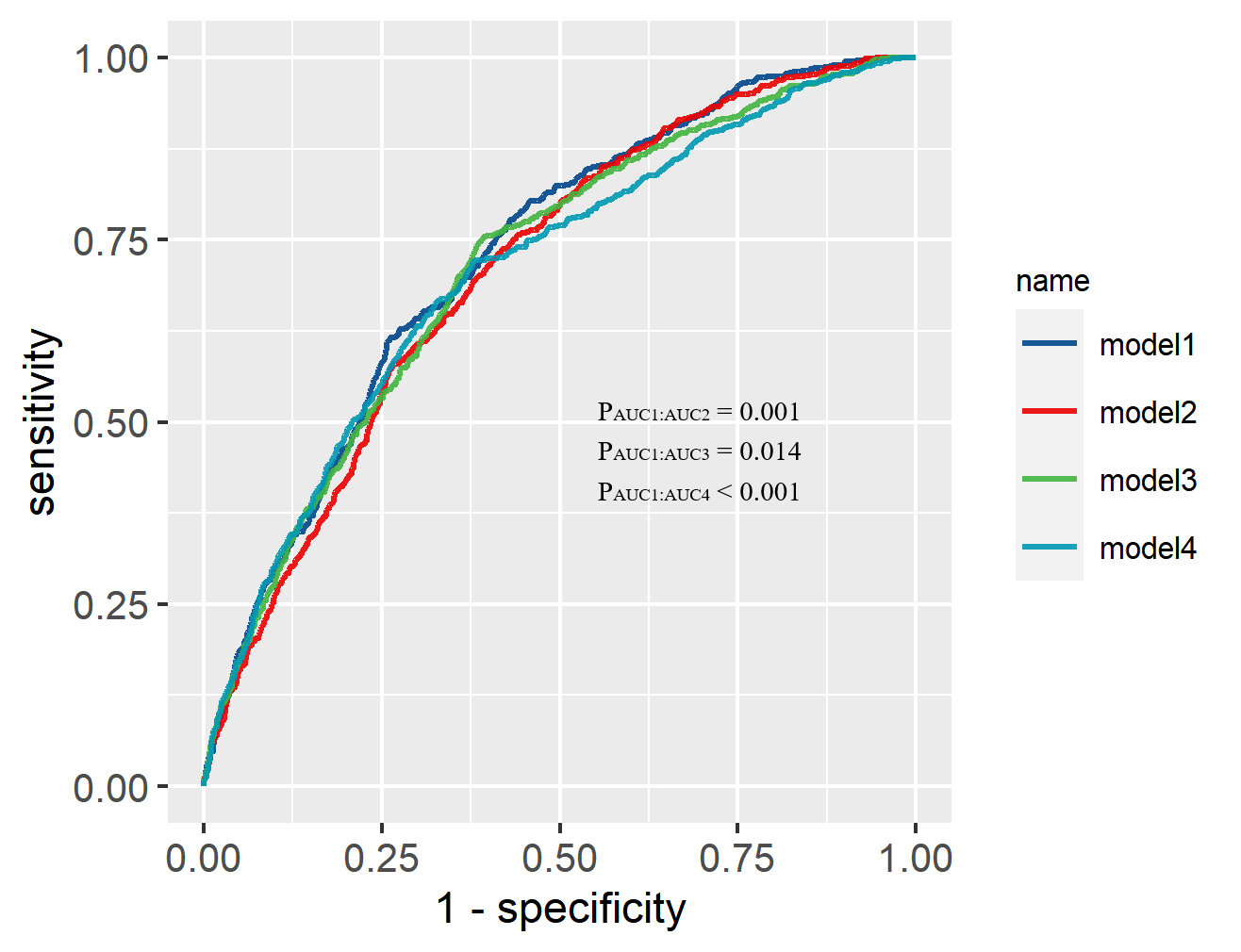
**

# eFigure 2. ROC curves between four Cox proportional hazards models. P-value indicates the significance level from the comparison of the AUC difference between the two ROC curves

**
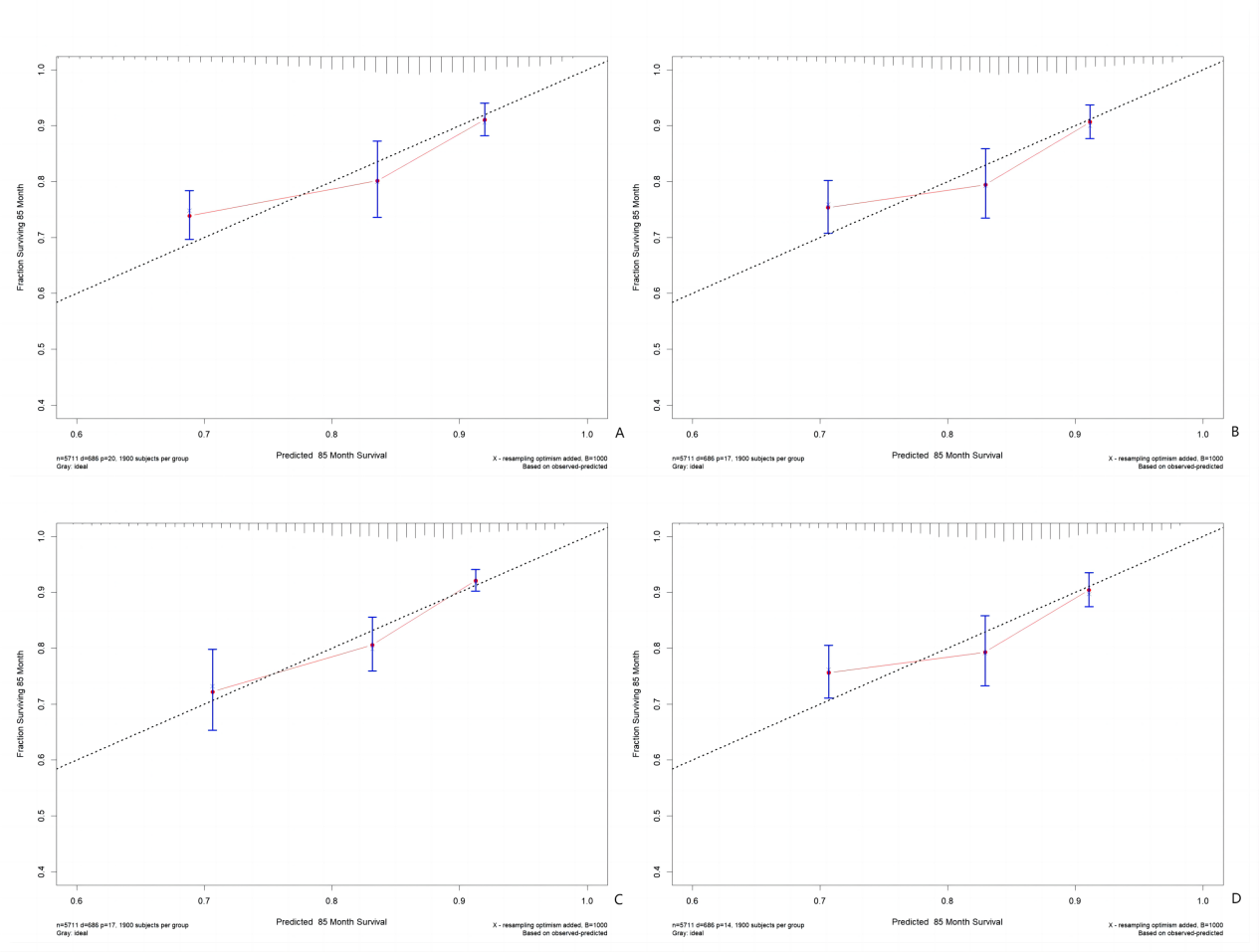
**

# eFigure 3. Calibration plots of four Cox proportional hazards models. A, Model 1. B, Model 2. C, Model 3. D, Model 4

**
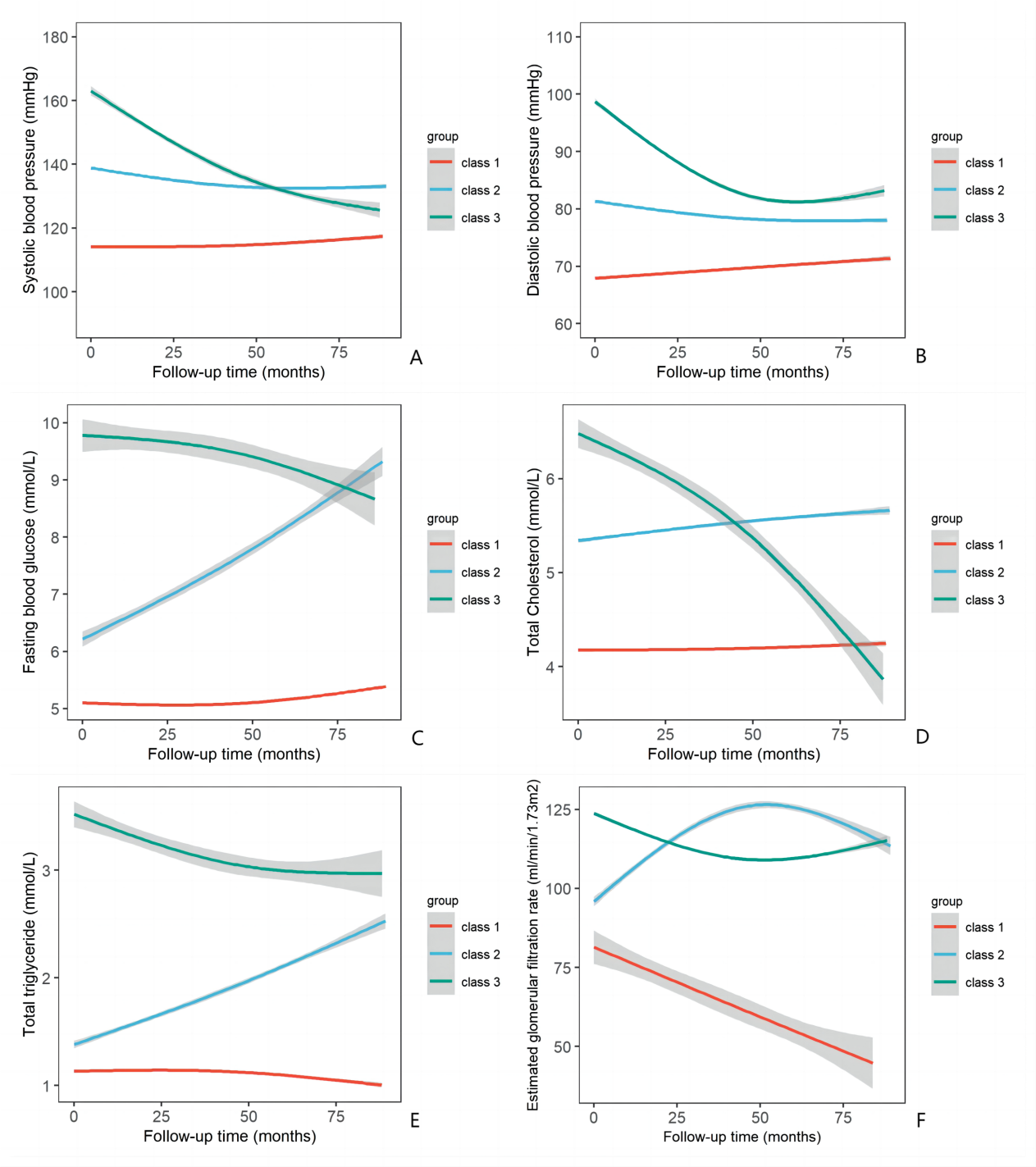
**eFigure 4. Trajectories of BP and metabolic biomarkers predicted by the LCGMM without fitting age and sex among 5711 disabled individuals. A, SBP, B, DBP, C, FBG. D, TG. E, TG. F, eGFR. The solid line represents the average blood pressure in a class and the shaded area indicated 95% CIs

**
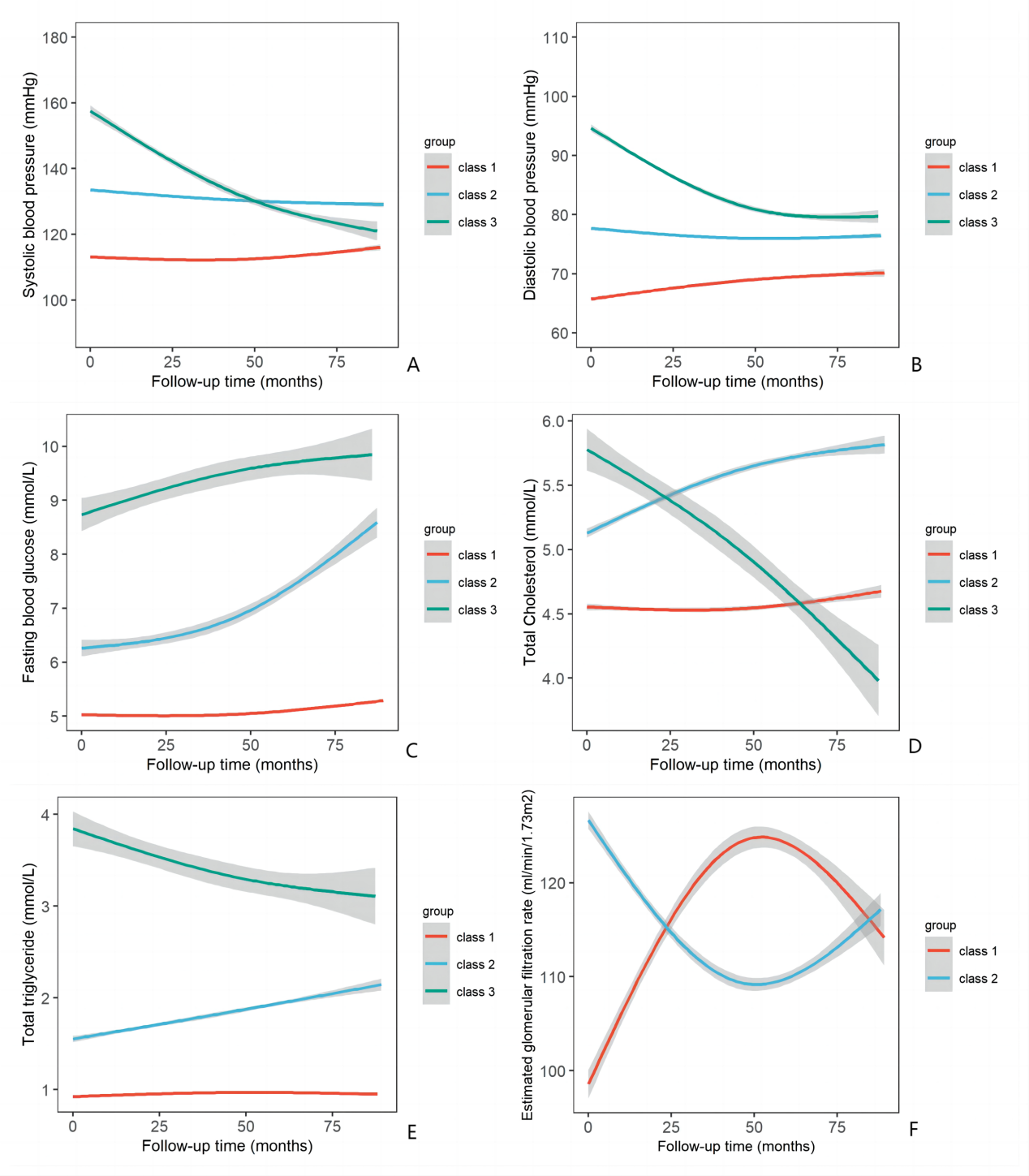
**

# eFigure 5. Trajectories of BP and metabolic biomarkers predicted by the LCGMM among 1528 disabled individuals with hypertension at baseline. A, SBP, B, DBP, C, FBG. D, TG. E, TG. F, eGFR. The solid line represents the average blood pressure in a class and the shaded area indicated 95% CIs

**
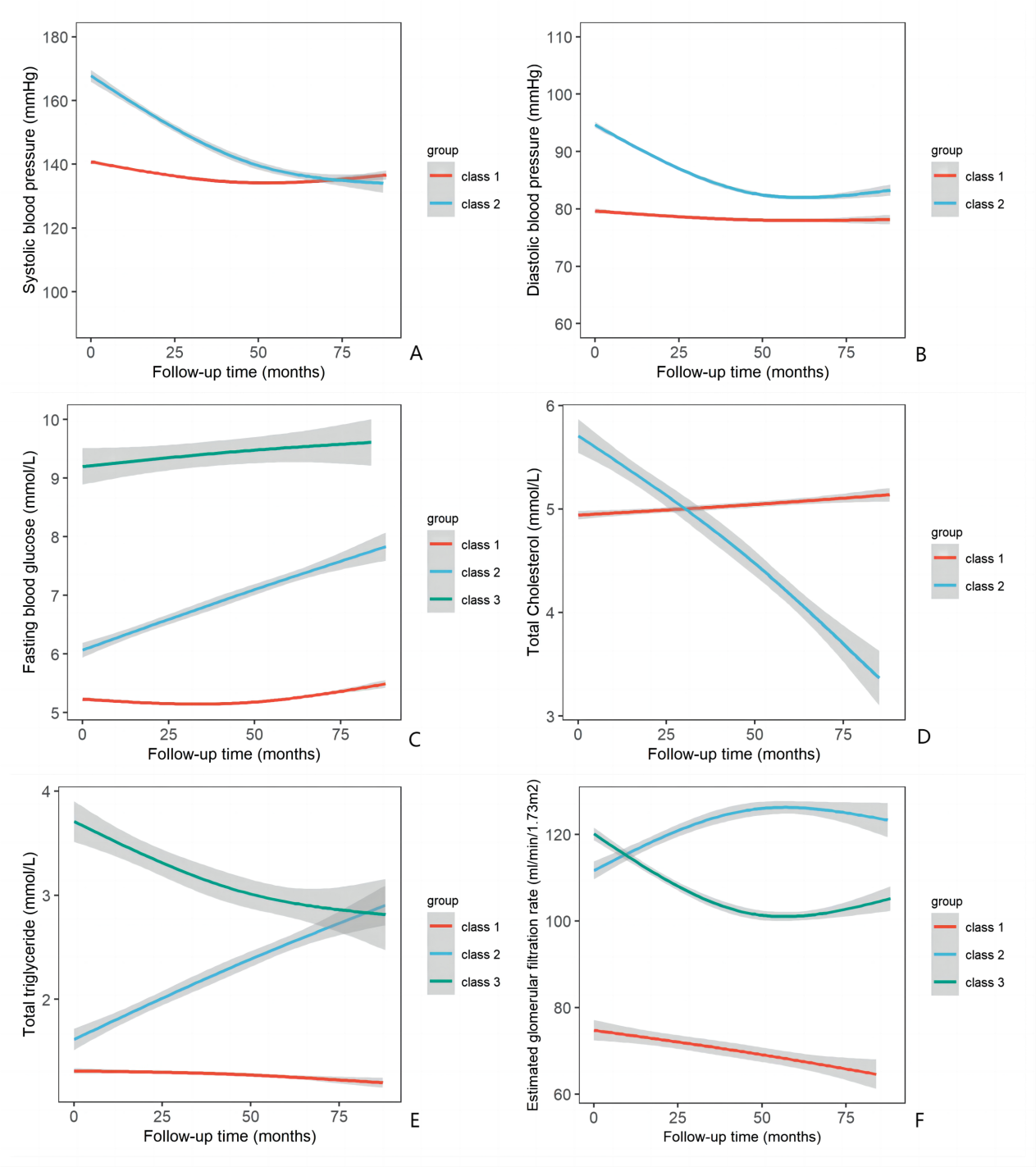
**

# eFigure 6. Trajectories of BP and metabolic biomarkers predicted by the LCGMM among 4183 disabled individuals without hypertension at baseline. A, SBP, B, DBP, C, FBG. D, TG. E, TG. F, eGFR. The solid line represents the average blood pressure in a class and the shaded area indicated 95% CIs

# eTable 1. Baseline characteristics of 5711 disabilities with CHD and No-CHD group

| Characteristics | Overall *n*=5711 | CHD group  *n*=686 | No-CHD group  *n*=5025 | *P*-value |
| --- | --- | --- | --- | --- |
| Age, yrs | 49.03(54.49,58.55) | 52.50(56.10,59.47) | 46.87(53.34,57.83) | **<0.001** |
| Female, *n* (%) | 2759(48.31) | 356(51.90) | 2403(47.82) | **0.045** |
| Education, *n* (%) |  |  |  | **0.003** |
| Primary school & Illiterate | 1092(19.12) | 103(15.01) | 989(19.68) |  |
| Junior high school | 4317(75.59) | 535(77.99) | 3782(75.26) |  |
| Senior high school & Higher | 302(5.29) | 48(7.00) | 254(5.05) |  |
| BMI, kg/m^2^ | 24.05±3.50 | 24.46±3.45 | 24.00±3.51 | **0.001** |
| Classification of disabilities, *n* (%) |  |  |  | **<0.001** |
| Intellectual & mental disability | 949(16.62) | 73(10.64) | 876(17.43) |  |
| Hearing & speech disability | 342(5.99) | 35(5.10) | 307(6.11) |  |
| Visual disability | 1230(21.54) | 169(24.64) | 1061(21.11) |  |
| Physical disability | 3190(55.86) | 409(59.62) | 2781(55.34) |  |
| Grading of disabilities, *n* (%) |  |  |  | 0.593 |
| Very severe disability | 437(7.65) | 45(6.56) | 392(7.80) |  |
| Severe disability | 883(15.46) | 114(16.62) | 769(15.30) |  |
| Moderate disability | 1910(33.44) | 228(33.24) | 1682(33.47) |  |
| Mild disability | 2481(43.44) | 299(43.59) | 2182(43.42) |  |
| Comorbidities, *n* (%) |  |  |  |  |
| Hypertension | 1528(26.76) | 285(41.55) | 1243(24.74) | **<0.001** |
| Diabetes mellitus | 694(12.15) | 122(17.78) | 572(11.38) | **<0.001** |
| hyperlipemia | 943(16.51) | 115(16.76) | 828(16.48) | 0.896 |
| Fatty liver disease | 539(9.44) | 55(8.02) | 484(9.63) | 0.096 |
| Chronic kidney disease | 532(9.32) | 72(10.50) | 460(9.15) | 0.114 |
| Resting heart rate, beats/min | 76(80,84) | 72(76,84) | 72(76,84) | 0.416 |
| Blood pressure, mmHg |  |  |  |  |
| SBP | 132.49±19.99 | 134.50±20.54 | 132.21±19.90 | **0.005** |
| DBP | 78.88±12.31 | 79.70±12.63 | 78.77±12.26 | 0.064 |
| Metabolic biomarkers |  |  |  |  |
| AFP, ng/mL | 0.48(0.95,1.79) | 0.47(0.95,1.70) | 0.43(0.90,1.70) | 0.310 |
| CEA, ng/ml | 0.53(1.19,2.42) | 0.53(1.12,2.24) | 0.49(1.06,2.08) | 0.190 |
| Hb, g/L | 138.69±15.98 | 137.40±15.59 | 138.89±16.03 | **0.013** |
| RBC, 10^12^/L | 4.55±0.45 | 4.52±0.44 | 4.56±0.45 | **0.017** |
| WBC, 10^9^/L | 6.31±1.64 | 6.47±1.94 | 6.28±1.59 | **0.002** |
| PLT, 10^9^/L | 201.35±54.53 | 203.37±55.37 | 201.05±54.40 | 0.258 |
| FBG, mmol/L | 5.00(5.40,6.10) | 4.90(5.20,5.70) | 4.80(5.20,5.60) | **0.016** |
| TC, mmol/L | 4.83±0.93 | 4.93±0.99 | 4.81±0.92 | **0.001** |
| TG, mmol/L | 1.05(1.47,2.18) | 1.01(1.32,1.97) | 0.87(1.24,1.82) | **<0.001** |
| Glo, g/L | 28.40±3.77 | 28.54±3.75 | 28.38±3.78 | 0.269 |
| Alt, U/L | 17.00(24.00,35.25) | 15.00(21.00,30.00) | 15.00(20.00,29.00) | 0.546 |
| TP, g/L | 72.25±4.08 | 72.09±3.97 | 72.27±4.10 | 0.247 |
| Alb, g/L | 43.85±2.37 | 43.57±2.34 | 43.89±2.37 | **<0.001** |
| UA, μmol/L | 322.70±85.82 | 329.60±85.53 | 321.69±85.82 | **0.014** |
| SU, mmol/L | 5.14±1.34 | 5.31±1.33 | 5.11±1.34 | **<0.001** |
| SCr, μmol/L | 51.38(62.65,75.30) | 50.20(60.75,73.90) | 50.50(61.60,72.30) | 0.924 |
| eGFR, ml/min/1.73 m^2^ | 91.63(110.98,131.93) | 91.96(108.66,129.94) | 95.36(113.07,135.07) | **<0.001** |

Abbreviation: BMI, body mass index; AFP, alpha fetoprotein; CEA, carcinoembryonic antigen; FBG, fasting plasma glucose; TC, total cholesterol; TG, total triglyceride; TP, total protein; Alb, albumin; Glo, globulin; Alt, alanine aminotransferase; UA, uric acid; SCr, serum creatinine; SU, serum urea; Hb, hemoglobin; RBC, red blood count; WBC, white blood count; PLT, platelet count; eGFR, estimated glomerular filtration rate.

# eTable 2. BIC values for different link functions and degrees of different variables

| Link function | Degree | Number of latent classes | | | |
| --- | --- | --- | --- | --- | --- |
|  |  | 2 | 3 | 4 | 5 |
| SBP |  |  |  |  |  |
| Linear | 1 | 24693.93 | 24649.59 | 25087.57 | 24677.70 |
|  | 2 | 24487.66 | 24441.04 | 24617.20 | 25049.95 |
|  | 3 | 24477.41 | 24493.57 | 24573.23 | 24956.05 |
| Beta | 1 | 24319.94 | 24272.87 | 24297.25 | 24498.64 |
|  | 2 | 24176.84 | **24145.76** | 24250.82 | 24628.76 |
|  | 3 | 24177.53 | 24158.68 | 24290.84 | 24775.08 |
| Splines | 1 | 24292.63 | 24255.65 | 24317.49 | 24489.57 |
|  | 2 | **24146.86** | **24127.65** | 24185.02 | 24378.71 |
|  | 3 | **24147.37** | **24139.48** | 24208.39 | 24862.49 |
| DBP |  |  |  |  |  |
| Linear | 1 | 21746.29 | 21793.87 | 21590.13 | 21684.81 |
|  | 2 | 21516.65 | 21350.65 | **21326.33** | 21588.61 |
|  | 3 | 21528.44 | 21504.54 | 21345.91 | 21945.47 |
| Beta | 1 | 21587.44 | 21499.71 | 21513.37 | 21545.94 |
|  | 2 | 21407.47 | **21183.82** | **21319.03** | 21785.89 |
|  | 3 | 21421.00 | 21476.58 | **21315.23** | 21560.76 |
| Splines | 1 | 21499.78 | 21740.85 | 22061.55 | 21608.58 |
|  | 2 | 22155.09 | 21595.50 | 22172.58 | 22291.90 |
|  | 3 | **21278.68** | 22287.45 | 22273.29 | 21538.84 |
| FBG |  |  |  |  |  |
| Linear | 1 | 84095.15 | 80853.87 | 79774.71 | 79768.53 |
|  | 2 | 83630.13 | 80720.73 | 78648.83 | 80759.23 |
|  | 3 | 83525.91 | 80592.96 | 78237.81 | 80852.28 |
| Beta | 1 | 53932.49 | 53338.16 | 52921.69 | 53034.90 |
|  | 2 | 53595.69 | 53028.26 | 53484.11 | 53018.46 |
|  | 3 | 53287.40 | 52825.52 | 52746.66 | 52463.43 |
| Splines | 1 | 52104.81 | 51884.09 | 51796.49 | 52017.33 |
|  | 2 | 51608.55 | **51396.21** | 51489.66 | 51633.66 |
|  | 3 | **51444.49** | **51262.12** | **51273.29** | **51357.60** |
| TG |  |  |  |  |  |
| Linear | 1 | 79509.57 | 77657.53 | 78063.41 | 75996.53 |
|  | 2 | 79419.51 | 76582.49 | 77894.60 | 76846.72 |
|  | 3 | 79520.11 | 78474.26 | 75481.89 | 77408.28 |
| Beta | 1 | 45744.08 | **45714.50** | **45646.71** | **45626.91** |
|  | 2 | 45744.51 | **45422.94** | 45875.54 | 45934.19 |
|  | 3 | 45746.48 | 45892.51 | **45696.31** | 45940.68 |
| Splines | 1 | 60846.05 | 60949.65 | 60869.67 | 61021.95 |
|  | 2 | 60819.51 | 60828.54 | 60743.57 | 61172.93 |
|  | 3 | 60665.72 | 60678.25 | 60936.43 | 60783.88 |
| TC |  |  |  |  |  |
| Linear | 1 | 79509.57 | 77657.53 | 78063.41 | 75996.53 |
|  | 2 | 79419.51 | 76582.49 | 77894.60 | 76846.72 |
|  | 3 | 79520.11 | 78474.26 | 75481.89 | 77408.28 |
| Beta | 1 | 61039.76 | 61120.56 | 61151.75 | 61108.18 |
|  | 2 | 61065.18 | 61018.65 | 60885.39 | 60883.70 |
|  | 3 | 61047.05 | **60739.72** | 60982.93 | 61216.82 |
| Splines | 1 | 60846.05 | 60949.65 | 60869.67 | 61021.95 |
|  | 2 | 60819.51 | 60828.54 | **60678.25** | 61172.93 |
|  | 3 | **60743.57** | **60665.72** | 60936.43 | **60783.88** |
| eGFR |  |  |  |  |  |
| Linear | 1 | 91960.09 | 91783.79 | 91636.80 | 91740.22 |
|  | 2 | 91944.20 | 91758.66 | 91719.76 | 91899.80 |
|  | 3 | 91951.02 | 91938.49 | 91819.07 | 92274.66 |
| Beta | 1 | 87525.52 | 87173.14 | 87133.90 | 87278.09 |
|  | 2 | 87488.63 | **86699.20** | **86701.87** | **86502.08** |
|  | 3 | 87473.80 | **86482.17** | 87563.47 | 87072.26 |
| Splines | 1 | 87511.83 | 87517.16 | 87088.86 | 87472.03 |
|  | 2 | 87420.07 | **86706.63** | 87477.46 | 87449.75 |
|  | 3 | 87483.51 | 87739.71 | 87539.36 | 87533.60 |

# eTable 3. Posterior probabilities of latent classes with different variables

| Model | BIC | Average posterior probabilities in each class | Posterior probabilities > 0.7 (%) | Minimum subgroup (%) |
| --- | --- | --- | --- | --- |
| SBP |  |  |  |  |
| Model 1 | 24127.65 | 0.72, 0.71, 0.74 | 62.83, 64.80, 66.40 | 8.12 |
| Model 2 | 24139.48 | **0.73, 0.71, 0.72** | **75.01, 65.50, 72.45** | **8.53** |
| Model 3 | 24145.76 | 0.73, 0.73, 0.73 | 52.54, 61.01, 56.15 | 7.84 |
| Model 4 | 24146.86 | 0.75, 0.89 | 59.04, 88.22 | 17.83 |
| Model 5 | 24147.37 | 0.75, 0.89 | 65.02, 88.53 | 17.56 |
| DBP |  |  |  |  |
| Model 1 | 21183.82 | **0.77, 0.74, 0.79** | **72.07, 68.40, 69.37** | **9.35** |
| Model 2 | 21278.68 | 0.82, 0.84 | 74.10, 79.33 | 44.42 |
| Model 3 | 21315.23 | 0.75, 0.72, 0.78, 0.79 | 59.34, 50.00, 72.81, 70.18 | 0.38 |
| Model 4 | 21319.03 | 0.75, 1.00, 0.77, 0.79 | 58.49, 100.00, 71.96, 69.60 | 0.21 |
| Model 5 | 21326.33 | 0.77, 0.95, 0.78, 0.80 | 65.78, 100.00, 72.50, 69.36 | 0.04 |
| FBG |  |  |  |  |
| Model 1 | 51262.12 | **0.90, 0.76, 0.81** | **91.82, 66.13, 70.23** | **6.71** |
| Model 2 | 51273.29 | 0.82, 0.80, 0.90, 0.68 | 74.37, 60.00, 91.11, 45.17 | 0.18 |
| Model 3 | 51357.60 | 0.75, 0.00, 0.00, 0.83, 0.00 | 61.23, 0.00, 0.00, 79.88, 0.00 | 0.00 |
| Model 4 | 51396.21 | 0.73, 0.71, 0.72 | 54.69, 56.68, 52.96 | 7.65 |
| Model 5 | 51444.49 | 0.75, 0.89 | 60.02, 88.53 | 17.56 |
| TC |  |  |  |  |
| Model 1 | 60665.72 | 0.81, 0.64, 0.63 | 71.55, 66.49, 63.74 | 5.39 |
| Model 2 | 60678.25 | 0.77, 0.83, 0.62, 0.64 | 64.47, 77.78, 62.08, 65.56 | 0.16 |
| Model 3 | 60739.72 | **0.82, 0.74, 0.73** | **74.15, 67.95, 73.81** | **5.38** |
| Model 4 | 60743.57 | 0.85, 0.99 | 79.65, 99.01 | 4.04 |
| Model 5 | 60783.88 | 0.74, 0.76, 0.61, 0.64, 0.63 | 59.62, 60.00, 59.41, 66.03, 56.61 | 0.30 |
| TG |  |  |  |  |
| Model 1 | 45422.94 | **0.74, 0.79, 0.72** | **72.72, 69.77, 68.04** | **13.38** |
| Model 2 | 45626.91 | 0.64, 0.64, 0.38, 0.00, 0.58 | 39.04, 37.42, 0.00, 0.00, 21.60 | 0.00 |
| Model 3 | 45646.71 | 0.60, 0.51, 0.54, 0.54 | 61.14, 54.17, 50.44, 53.54 | 10.40 |
| Model 4 | 45696.31 | 0.64, 0.50, 0.64, 0.56 | 66.97, 55.37, 63.87, 56.87 | 7.18 |
| Model 5 | 45714.5 | 0.66, 0.76, 0.63 | 65.31, 66.54, 63.21 | 5.60 |
| eGFR |  |  |  |  |
| Model 1 | 86482.17 | **0.89, 0.90, 0.81** | **87.50, 89.55, 71.69** | **5.66** |
| Model 2 | 86502.08 | 0.95, 0.86, 0.69, 0.77, 0.80 | 90.00, 73.17, 52.43, 64.02, 80.40 | 0.89 |
| Model 3 | 86699.20 | 0.86, 0.88, 0.80 | 80.65, 86.37, 68.47 | 5.09 |
| Model 4 | 86701.87 | 0.97, 0.89, 0.84, 0.79 | 95.73, 84.62, 81.20, 67.43 | 0.77 |
| Model 5 | 86706.63 | 0.74, 0.84, 0.72 | 68.30, 79.55, 61.76 | 6.11 |

Note: P-values for all parameters in the LCGMMs fitted by the maximum likelihood method were less than 0.05.

# eTable 4. Longitudinal characteristics during follow-up of 5711 disabilities by BP trajectory cluster

| Characteristics | SBP | | | | DBP | | | |
| --- | --- | --- | --- | --- | --- | --- | --- | --- |
|  | Class 1 *n*=1956 | Class 2 *n*=3268 | Class 3 *n*=487 | *P*-value | Class 1 *n*=1671 | Class 2 *n*=3506 | Class 3 *n*=534 | *P*-value |
| Blood pressure |  |  |  |  |  |  |  |  |
| Average SBP, mmHg | 115.03±7.90 | 134.26±9.29 | 144.35±11.64 | **<0.001** | 116.14±9.97 | 131.90±10.40 | 145.20±11.12 | **<0.001** |
| Average DBP, mmHg | 70.65±5.47 | 79.77±5.74 | 84.54±6.18 | **<0.001** | 68.86±4.42 | 79.37±4.72 | 87.49±5.56 | **<0.001** |
| CV of SBP | 0.08±0.03 | 0.08±0.03 | 0.14±0.04 | **<0.001** | 0.08±0.03 | 0.08±0.04 | 0.11±0.05 | **<0.001** |
| CV of DBP | 0.09±0.04 | 0.09±0.04 | 0.12±0.04 | **<0.001** | 0.09±0.04 | 0.09±0.03 | 0.12±0.04 | **<0.001** |
| Metabolic biomarkers |  |  |  |  |  |  |  |  |
| Average FBG, mmol/L | 5.27±1.10 | 5.67±1.39 | 6.12±1.86 | **<0.001** | 5.30±1.21 | 5.63±1.35 | 6.06±1.76 | **<0.001** |
| Average TC, mmol/L | 4.86±0.81 | 4.99±0.86 | 5.06±0.85 | **<0.001** | 4.86±0.83 | 4.98±0.84 | 5.06±0.89 | **<0.001** |
| Average TG, mmol/L | 1.43±0.89 | 1.71±1.14 | 1.91±1.14 | **<0.001** | 1.39±0.85 | 1.72±1.15 | 1.85±1.04 | **<0.001** |
| Average eGFR, ml/min/1.73 m^2^ | 114.18±23.49 | 113.77±25.85 | 112.69±30.25 | 0.508 | 115.51±24.49 | 113.58±25.57 | 110.10±27.40 | **<0.001** |
| CV of FBG | 0.07±0.06 | 0.09±0.07 | 0.10±0.09 | **<0.001** | 0.07±0.06 | 0.08±0.07 | 0.10±0.09 | **<0.001** |
| CV of TC | 0.10±0.04 | 0.10±0.05 | 0.10±0.05 | **0.007** | 0.10±0.04 | 0.10±0.05 | 0.10±0.06 | 0.128 |
| CV of TG | 0.27±0.13 | 0.27±0.14 | 0.28±0.14 | 0.243 | 0.27±0.13 | 0.28±0.14 | 0.27±0.14 | 0.196 |
| CV of eGFR | 0.13±0.07 | 0.13±0.07 | 0.14±0.07 | **0.001** | 0.13±0.06 | 0.13±0.07 | 0.14±0.07 | 0.082 |
| FBG trajectories |  |  |  | **<0.001** |  |  |  | **<0.001** |
| Class 1 | 1767(90.34) | 2603(79.65) | 338(69.40) |  | 1497(89.59) | 2843(81.09) | 368(68.91) |  |
| Class 2 | 114(5.83) | 418(12.79) | 88(18.07) |  | 96(5.75) | 425(12.12) | 99(18.54) |  |
| Class 3 | 75(3.83) | 247(7.56) | 61(12.53) |  | 78(4.67) | 238(6.79) | 67(12.55) |  |
| TC trajectories |  |  |  | **<0.001** |  |  |  | **<0.001** |
| Class 1 | 1183(60.48) | 1904(58.26) | 298(61.19) |  | 1029(61.58) | 2048(58.41) | 308(57.68) |  |
| Class 2 | 706(36.09) | 1169(35.77) | 144(29.57) |  | 584(34.95) | 1261(35.97) | 174(32.58) |  |
| Class 3 | 67(3.43) | 195(5.97) | 45(9.24) |  | 58(3.47) | 197(5.62) | 52(9.74) |  |
| TG trajectories |  |  |  | **<0.001** |  |  |  | **<0.001** |
| Class 1 | 1354(69.22) | 2005(61.35) | 263(54.00) |  | 1190(71.21) | 2135(60.90) | 297(55.62) |  |
| Class 2 | 432(22.09) | 767(23.47) | 126(25.87) |  | 351(21.01) | 847(24.16) | 127(23.78) |  |
| Class 3 | 170(8.69) | 496(15.18) | 98(20.12) |  | 130(7.78) | 524(14.95) | 110(20.60) |  |
| eGFR trajectories |  |  |  | **<0.001** |  |  |  | **<0.001** |
| Class 1 | 80(4.09) | 194(5.94) | 49(10.06) |  | 54(3.23) | 218(6.22) | 51(9.55) |  |
| Class 2 | 772(39.47) | 1330(40.70) | 176(36.14) |  | 697(41.71) | 1379(39.33) | 202(37.83) |  |
| Class 3 | 1104(56.44) | 1744(53.37) | 262(53.80) |  | 920(55.06) | 1909(54.45) | 281(52.62) |  |

Abbreviation: FBG, fasting plasma glucose; TC, total cholesterol; TG, total triglyceride; eGFR, estimated glomerular filtration rate.

# eTable 5. Longitudinal characteristics during follow-up of 5711 disabilities with CHD and no-CHD group

| Characteristics | Overall *n*=5711 | CHD group  *n*=686 | No-CHD group  *n*=5025 | *P*-value |
| --- | --- | --- | --- | --- |
| Blood pressure |  |  |  |  |
| Average SBP, mmHg | 128.53±13.60 | 130.29±13.64 | 128.29±13.57 | **<0.001** |
| Average DBP, mmHg | 77.05±7.44 | 77.38±7.50 | 77.01±7.43 | 0.215 |
| CV of SBP | 0.09±0.04 | 0.09±0.04 | 0.09±0.04 | **0.002** |
| CV of DBP | 0.09±0.04 | 0.10±0.04 | 0.09±0.04 | **0.006** |
| SBP trajectories |  |  |  | **<0.001** |
| Class 1 | 1956(34.25) | 170(24.78) | 1786(35.54) |  |
| Class 2 | 3268(57.22) | 421(61.37) | 2847(56.66) |  |
| Class 3 | 487(8.53) | 95(13.85) | 392(7.80) |  |
| DBP trajectories |  |  |  | **<0.001** |
| Class 1 | 1671(29.26) | 164(23.91) | 1507(29.99) |  |
| Class 2 | 3506(61.39) | 430(62.68) | 3076(61.21) |  |
| Class 3 | 534(9.35) | 92(13.41) | 442(8.80) |  |
| Metabolic biomarkers |  |  |  |  |
| Average FBG, mmol/L | 5.57±1.37 | 5.81±1.67 | 5.54±1.32 | **<0.001** |
| Average TC, mmol/L | 4.95±0.84 | 4.95±0.87 | 4.95±0.84 | 0.827 |
| Average TG, mmol/L | 1.63±1.07 | 1.67±0.98 | 1.63±1.08 | 0.348 |
| Average eGFR, ml/min/1.73 m^2^ | 114.18±23.49 | 113.77±25.85 | 112.69±30.25 | 0.508 |
| CV of FBG | 0.08±0.07 | 0.09±0.08 | 0.08±0.07 | **<0.001** |
| CV of TC | 0.10±0.05 | 0.12±0.07 | 0.10±0.05 | **<0.001** |
| CV of TG | 0.27±0.14 | 0.27±0.14 | 0.27±0.14 | 0.465 |
| CV of eGFR | 0.13±0.07 | 0.14±0.08 | 0.13±0.07 | **0.002** |
| FBG trajectories |  |  |  | **<0.001** |
| Class 1 | 4708(82.44) | 520(75.80) | 4188(83.34) |  |
| Class 2 | 620(10.86) | 102(14.87) | 518(10.31) |  |
| Class 3 | 383(6.71) | 64(9.33) | 319(6.35) |  |
| TC trajectories |  |  |  | **0.012** |
| Class 1 | 3385(59.27) | 419(61.08) | 2966(59.02) |  |
| Class 2 | 2019(35.35) | 217(31.63) | 1802(35.86) |  |
| Class 3 | 307(5.38) | 50(7.29) | 257(5.11) |  |
| TG trajectories |  |  |  | 0.068 |
| Class 1 | 3622(63.42) | 446(65.01) | 3176(63.20) |  |
| Class 2 | 1325(23.20) | 137(19.97) | 1188(23.64) |  |
| Class 3 | 764(13.38) | 103(15.01) | 661(13.15) |  |
| eGFR trajectories |  |  |  | **0.038** |
| Class 1 | 323(5.66) | 50(7.29) | 273(5.43) |  |
| Class 2 | 2278(39.89) | 288(41.98) | 1990(39.60) |  |
| Class 3 | 3110(54.46) | 348(50.73) | 2762(54.97) |  |

Abbreviation: FBG, fasting plasma glucose; TC, total cholesterol; TG, total triglyceride; eGFR, estimated glomerular filtration rate.

# eTable 6. Cox regression model for BP trajectories and CHD risk in the disabled population

| Variables | Univariate cox model 1 | | Multivariate cox model 1 | |
| --- | --- | --- | --- | --- |
|  | HR (95% CI) | *P*-value | aHR (95% CI) | *P*-value |
| Age | 1.060(1.050,1.070) | **<0.001** | 1.054(1.042,1.067) | **<0.001** |
| Female | 1.158(0.997,1.345) | 0.055 | 1.050(1.037,1.062) | **0.001** |
| Education |  | **0.002** |  | **0.001** |
| Primary school & Illiterate | Reference | – | Reference | – |
| Junior high school | 1.324(1.072,1.634) | **0.009** | 1.218(0.981,1.513) | 0.075 |
| Senior high school & Higher | 1.819(1.292,2.562) | **0.001** | 1.745(1.232,2.460) | **0.002** |
| Classification of disabilities |  | **<0.001** |  | **<0.001** |
| Intellectual & mental disability | 0.589(0.459,0.755) | **<0.001** | 0.600(0.468,0.770) | **<0.001** |
| Hearing & speech disability | 0.807(0.571,1.140) | 0.223 | 0.831(0.588,1.174) | 0.294 |
| Visual disability | 1.079(0.902,1.291) | 0.405 | 1.084(0.906,1.298) | 0.376 |
| Physical disability | Reference | – | Reference | – |
| Grading of disabilities |  | 0.671 | – | – |
| Very severe disability | 1.244(0.881,1.756) | 0.216 | – | – |
| Severe disability | 1.156(0.840,1.592) | 0.373 | – | – |
| Moderate disability | 1.156(0.845,1.582) | 0.364 | – | – |
| Mild disability | Reference | – | – | – |
| Comorbidities |  |  |  |  |
| Hypertension (Yes) | 2.077(1.785,2.418) | **<0.001** | 1.475(1.240,1.756) | **<0.001** |
| Diabetes mellitus (Yes) | 1.643(1.351,1.999) | **<0.001** | 1.388(1.056,1.825) | **0.019** |
| Hyperlipemia (Yes) | 1.014(0.830,1.239) | 0.893 | – | – |
| Fatty liver disease (Yes) | 1.200(0.911,1.581) | 0.195 | – | – |
| Chronic kidney disease (Yes) | 1.047(0.820,1.337) | 0.713 | – | – |
| Blood pressure |  |  |  |  |
| SBP at baseline | 1.000(1.000,1.010) | **0.009** | 1.005(1.001,1.009) | **0.011** |
| DBP at baseline | 1.010(0.999,1.010) | 0.084 | 0.997(0.987,1.008) | 0.616 |
| SBP trajectories |  | **<0.001** |  | **0.017** |
| Class 1 | Reference | – | Reference | – |
| Class 2 | 1.560(1.305,1.864) | **<0.001** | 1.266(1.014,1.581) | **0.037** |
| Class 3 | 2.376(1.849,3.054) | **<0.001** | 1.609(1.157,2.238) | **0.005** |
| DBP trajectories |  | **<0.001** |  | 0.437 |
| Class 1 | Reference | – | Reference | – |
| Class 2 | 1.271(1.061,1.521) | **<0.001** | 0.929(0.719,1.199) | 0.570 |
| Class 3 | 1.796(1.391,2.318) | **<0.001** | 1.062(0.764,1.475) | 0.721 |
| Metabolic biomarkers |  |  |  |  |
| FBG at baseline | 1.090(1.050,1.140) | **<0.001** | 1.029(0.966,1.095) | 0.380 |
| TC at baseline | 1.150(1.070,1.250) | **<0.001** | 1.073(0.986,1.168) | 0.103 |
| TG at baseline | 1.040(0.988,1.090) | 0.140 | – | – |
| eGFR at baseline | 0.997(0.995,0.999) | **0.003** | 0.997(0.995,1.000) | **0.039** |
| FBG trajectories |  | **<0.001** |  | **0.019** |
| Class 1 | Reference | – | Reference | – |
| Class 2 | 1.551(1.255,1.919) | **0.001** | 1.273(0.883,1.836) | 0.196 |
| Class 3 | 1.565(1.207,2.029) | **<0.001** | 1.369(1.095,1.711) | **0.006** |
| TC trajectories |  | **0.039** |  | 0.245 |
| Class 1 | Reference | – | Reference | – |
| Class 2 | 0.873(0.741,1.028) | 0.103 | 0.871(0.735,1.032) | 0.111 |
| Class 3 | 1.272(0.949,1.706) | 0.107 | 1.020(0.751,1.386) | 0.900 |
| TG trajectories |  | 0.059 |  | 0.481 |
| Class 1 | Reference | – | Reference | – |
| Class 2 | 0.826(0.682,1.001) | 0.051 | 0.887(0.729,1.080) | 0.234 |
| Class 3 | 1.105(0.892,1.369) | 0.360 | 0.947(0.759,1.182) | 0.632 |
| eGFR trajectories |  | **0.018** |  | **0.040** |
| Class 1 | 1.174(1.004,1.373) | **0.044** | 1.143(0.977,1.337) | 0.095 |
| Class 2 | Reference | – | Reference | – |
| Class 3 | 1.443(1.073,1.942) | **0.015** | 1.407(1.046,1.893) | **0.024** |

# eTable 7. Univariate Cox regression model for BP and CHD risk in the disabled population

| Variables | Univariate cox model 2^&^ | | Univariate cox model 3^$^ | | Univariate cox model 4^@^ | |
| --- | --- | --- | --- | --- | --- | --- |
|  | HR (95% CI) | *P*-value | HR (95% CI) | *P*-value | HR (95% CI) | *P*-value |
| Blood pressure | | | | | | |
| SBP | 1.010(1.004,1.015) | **0.001** | 18.053(2.633,123.795) | **0.003** | 1.000(1.000,1.010) | **0.009** |
| DBP | 1.006(0.996,1.016) | 0.252 | 11.459(1.739,75.506) | **0.011** | 1.010(0.999,1.010) | 0.084 |
| Metabolic biomarkers | | | | | | |
| FBG | 1.116(1.069,1.165) | **<0.001** | 5.850(2.518,13.592) | **<0.001** | 1.090(1.050,1.140) | **<0.001** |
| TC | 0.989(0.904,1.081) | 0.801 | 3.256(2.672,3.968) | **<0.001** | 1.150(1.070,1.250) | **<0.001** |
| TG | 1.034(0.969,1.103) | 0.312 | 0.816(0.466,1.429) | 0.476 | 1.040(0.988,1.090) | 0.140 |
| eGFR | 0.994(0.991,0.997) | **<0.001** | 2.621(1.062,6.471) | **0.037** | 0.997(0.995,0.999) | **0.003** |

^&^The average BP and metabolic biomarkers during follow-up were analyzed in model 2. ^$^The CVs of BP and metabolic biomarkers during follow-up were analyzed in model 3. ^@^The BP and metabolic biomarkers at baseline were analyzed in model 4.

# eTable 8. Multivariate Cox regression model for blood pressure and CHD risk in the disabled population

| Variables | Multivariate cox model 2^&^ | | Multivariate cox model 3^$^ | | Multivariate cox model 4^@^ | |
| --- | --- | --- | --- | --- | --- | --- |
|  | aHR (95% CI) | *P*-value | aHR (95% CI) | *P*-value | aHR (95% CI) | *P*-value |
| Age | 1.054(1.042,1.067) | **<0.001** | 1.053(1.041,1.065) | **<0.001** | 1.052(1.041,1.064) | **<0.001** |
| Female | 1.236(1.062,1.438) | **0.006** | 1.236(1.058,1.443) | **0.007** | 1.170(1.001,1.366) | **0.048** |
| Education |  | **0.004** |  | **0.010** |  | **0.005** |
| Primary school & Illiterate | Reference | – | Reference | – | Reference | – |
| Junior high school | 1.234(0.993,1.533) | 0.058 | 1.209(0.974,1.501) | 0.086 | 1.231(0.990,1.530) | 0.061 |
| Senior high school & Higher | 1.811(1.280,2.564) | **0.001** | 1.707(1.207,2.414) | **0.002** | 1.786(1.261,2.529) | **0.001** |
| Classification of disabilities |  | **0.008** |  | **<0.001** |  | **0.008** |
| Intellectual & mental disability | 0.661(0.512,0.854) | **0.002** | 0.618(0.479,0.798) | **<0.001** | 0.660(0.511,0.854) | **0.002** |
| Hearing & speech disability | 0.814(0.576,1.152) | 0.246 | 0.822(0.582,1.162) | 0.267 | 0.822(0.582,1.163) | 0.269 |
| Visual disability | 1.028(0.858,1.232) | 0.763 | 1.094(0.914,1.309) | 0.325 | 1.038(0.867,1.243) | 0.685 |
| Physical disability | Reference | – | Reference | – | Reference | – |
| Comorbidities |  |  |  |  |  |  |
| Hypertension (Yes) | 1.885(1.582,2.247) | **<0.001** | 1.618(1.379,1.899) | **<0.001** | 1.890(1.586,2.252) | **<0.001** |
| Diabetes mellitus (Yes) | 1.354(1.048,1.749) | **0.020** | 1.307(1.036,1.648) | **0.024** | 1.455(1.194,1.775) | **<0.001** |
| Blood pressure |  |  |  |  |  |  |
| SBP at baseline | 1.001(0.993,1.009) | 0.726 | 1.004(1.000,1.007) | 0.060 | 1.004(1.000,1.008) | **0.039** |
| DBP at baseline | 0.994(0.983,1.004) | 0.239 | 0.996(0.986,1.006) | 0.405 | 0.995(0.984,1.006) | 0.346 |
| SBP during follow-up | 1.006(1.001,1.012) | **0.032** | 10.454(1.407,77.666) | **0.004** | – | – |
| DBP during follow-up | – | – | 4.652(0.579,37.409) | 0.148 | – | – |
| Metabolic biomarkers |  |  |  |  |  |  |
| FBG at baseline | 0.987(0.907,1.075) | 0.768 | 1.061(1.008,1.117) | **0.025** | 1.091(1.046,1.139) | **<0.001** |
| TC at baseline | 1.029(0.947,1.119) | 0.499 | 1.084(1.000,1.175) | 0.051 | 1.082(0.998,1.173) | 0.056 |
| eGFR at baseline | 1.000(0.997,1.003) | 0.887 | 0.996(0.994,0.998) | **<0.001** | 0.997(0.995,0.999) | **0.005** |
| FBG during follow-up | 1.122(1.072,1.174) | **<0.001** | 2.977(1.019,8.694) | **0.046** | – | – |
| TC during follow-up | – | – | 2.833(2.285,3.511) | **<0.001** | – | – |
| eGFR during follow-up | 0.994(0.991,0.997) | **<0.001** | 4.822(1.592,14.609) | **0.005** | – | – |

^&^The average BP and metabolic biomarkers during follow-up were analyzed in model 2. ^$^The CVs of BP and metabolic biomarkers during follow-up were analyzed in model 3. ^@^The BP and metabolic biomarkers at baseline were analyzed in model 4.

# eTable 9. Sensitivity analysis of the correlation between BP-related indicators and the incidence of CHD in the disabled population

| Variables | Model 1^#^ | Model 2^&^ | Model 3^$^ | Model 4^@^ |
| --- | --- | --- | --- | --- |
| Without fitting age and sex (n=5711) | | | | |
| SBP trajectories |  |  |  |  |
| Class 1 | Reference |  |  |  |
| Class 2 | **1.279(1.029,1.588)**^*^ |  |  |  |
| Class 3 | **1.740(1.235,2.452)^*^** |  |  |  |
| DBP trajectories |  |  |  |  |
| Class 1 | Reference |  |  |  |
| Class 2 | 1.090(0.915,1.299) |  |  |  |
| Class 3 | 1.204(0.924,1.571) |  |  |  |
| SBP |  | **1.006(1.001,1.012)**^*^ | **10.454(1.407,77.666)^*^** | **1.004(1.000,1.008)**^*^ |
| DBP |  | – | 4.652(0.579,37.409) | 0.995(0.984,1.006) |
| With hypertension at baseline (n=1528) | | | | |
| SBP trajectories |  |  |  |  |
| Class 1 | Reference |  |  |  |
| Class 2 | **1.344(1.058,1.709)^*^** |  |  |  |
| DBP trajectories |  |  |  |  |
| Class 1 | Reference |  |  |  |
| Class 2 | 1.059(0.776,1.444) |  |  |  |
| SBP |  | **1.011(1.001,1.021)**^*^ | **22.474(1.069,472.508)^*^** | **1.011(1.002,1.019)**^*^ |
| DBP |  | 0.997(0.977,1.017) | 8.908(0.412,192.543) | 0.987(0.973,1.002) |
| Without hypertension at baseline (n=4183) | | | | |
| SBP trajectories |  |  |  |  |
| Class 1 | Reference |  |  |  |
| Class 2 | 0.944(0.721,1.236) |  |  |  |
| Class 3 | **1.797(1.073,3.011)**^*^ |  |  |  |
| DBP trajectories |  |  |  |  |
| Class 1 | Reference |  |  |  |
| Class 2 | 0.984(0.701,1.382) |  |  |  |
| Class 3 | 1.250(0.815,1.916) |  |  |  |
| SBP |  | **1.016(1.002,1.029)**^*^ | **15.792(1.074,232.162)^*^** | **1.010(1.001,1.019)**^*^ |
| DBP |  | - | 7.411(0.470,116.934) | 0.995(0.982,1.009) |

^#^The trajectories of BP and metabolic biomarkers during follow-up were analyzed in model 1. ^&^The average BP and metabolic biomarkers during follow-up were analyzed in model 2. ^$^The CVs of BP and metabolic biomarkers during follow-up were analyzed in model 3. ^@^The BP and metabolic biomarkers at baseline were analyzed in model 4. ^*^P values indicate significance levels less than 0.05 for BP-related indicators after adjustment for covariates.

# eTable 10. Sensitivity analysis for the comparison of evaluation indicators of the four Cox proportional hazards models

| Indexes | Model 1^#^ | Model 2^&^ | Model 3^$^ | Model 4^@^ |
| --- | --- | --- | --- | --- |
| Without fitting age and sex (n=5711) | | | | |
| Goodness-of-fit |  |  |  |  |
| AIC | 2306.32 | 2324.38 | 2310.18 | 2330.04 |
| BIC | 2379.47 | 2384.23 | 2383.33 | 2389.89 |
| Discrimination |  |  |  |  |
| AUC | 0.718^*^ | 0.709 | 0.713 | 0.706 |
| With hypertension at baseline (n=1528) | | | | |
| Goodness-of-fit |  |  |  |  |
| AIC | 1356.30 | 1367.03 | 1363.45 | 1371.16 |
| BIC | 1404.28 | 1404.36 | 1406.11 | 1408.49 |
| Discrimination |  |  |  |  |
| AUC | 0.716^*^ | 0.707 | 0.712 | 0.703 |
| Without hypertension at baseline (n=4183) | | | | |
| Goodness-of-fit |  |  |  |  |
| AIC | 1589.65 | 1598.53 | 1596.62 | 1604.92 |
| BIC | 1646.69 | 1642.90 | 1653.67 | 1655.63 |
| Discrimination |  |  |  |  |
| AUC | 0.705^*^ | 0.690 | 0.698 | 0.674 |

^#^The trajectories of BP and metabolic biomarkers during follow-up were analyzed in model 1. ^&^The average BP and metabolic biomarkers during follow-up were analyzed in model 2. ^$^The CVs of BP and metabolic biomarkers during follow-up were analyzed in model 3. ^@^The BP and metabolic biomarkers at baseline were analyzed in model 4. ^*^P-values indicate that the significance level of the difference between the AUC of model 1 and the other three models were less than 0.05.
